# Supplementary material for: The Magnetic Behaviour of CoTPP Supported on Coinage Metal Surfaces in the Presence of Small Molecules: A Molecular Cluster Study of the Surface trans-Effect
Source: Nanomaterials (Basel). 2022 Jan 10;12(2):218. doi: 10.3390/nano12020218 (PMC8778902; doi:10.3390/nano12020218)
Supplement: Supplementary file 1 [file nanomaterials-12-00218-s001.zip › nanomaterials-1509116-supplementary.pdf]

nanomaterials

MDPI

Supplementary Materials

The Magnetic Behaviour of CoTPP Supported on Coinage Metal Surfaces in the Presence of Small Molecules: A Molecular Cluster Study of the Surface *trans*-Effect

Silvia Carlotto [1,2,*], Iulia Cojocariu [3], Vitaliy Feyer [3,4], Luca Floreano [5] and Maurizio Casarin [1,2,*]

1 Dipartimento di Scienze Chimiche, Università degli Studi di Padova, Via Francesco Marzolo 1, 35131 Padova, Italy
2 Istituto di Chimica della Materia Condensata e di Tecnologie per l'Energia (ICMATE), Consiglio Nazionale delle Ricerche (CNR) presso Dipartimento di Scienze Chimiche, Università degli Studi di Padova, Via F. Marzolo 1, 35131 Padova, Italy
3 Peter Grünberg Institute (PGI-6), Forschungszentrum Jülich GmbH, Leo-Brandt-Straße, 52428 Jülich, Germany; i.cojocariu@fz-juelich.de (I.C.); v.feyer@fz-juelich.de (V.F.)
4 Fakultät f. Physik and Center for Nanointegration Duisburg-Essen (CENIDE), Universität Duisburg-Essen, D-47048 Duisburg, Germany
5 Istituto Officina dei Materiali (IOM), Consiglio Nazionale delle Ricerche (CNR), Lab. TASC, S.S. 14, Km. 163,5, 34149 Trieste, Italy; floreano@iom.cnr.it
* Correspondence: silvia.carlotto@unipd.it (S.C.); maurizio.casarin@unipd.it (M.C.)

Supplementary Materials

Table S1. Optimized coordinates (Å) of the free CO ($C_{\infty v}$) at the SR-ZORA, BP86, TZP, spin-restricted level of theory.

C	0.000000	0.000000	0.000000
O	0.000000	0.000000	1.139351

Table S2. Optimized coordinates (Å) of the free NH_3 (C_{3v}) at the SR-ZORA, BP86, TZP, spin-restricted level of theory.

N	0.000000	0.000000	0.000000
H	0.942499	0.000000	0.401378
H	−0.471250	0.816228	0.401378
H	−0.471250	−0.816228	0.401378

Table S3. Optimized coordinates (Å) of the free NO ($C_{\infty v}$) at the SR-ZORA, BP86, TZP, spin-unrestricted level of theory.

N	0.000000	0.000000	0.000000
O	0.000000	0.000000	1.165578

Table S4. Optimized coordinates (Å) of the free NO_2 (C_{2v}) at the SR-ZORA, BP86, TZP, spin-unrestricted level of theory.

N	0.000000	0.000000	0.000000
O	−1.114346	0.000000	−0.481995
O	1.114346	0.000000	−0.481995

Table S5. Optimized coordinates (Å) of the free O_2 ($D_{\infty h}$) at the SR-ZORA, BP86, TZP, spin-unrestricted level of theory.

O	0.000000	0.000000	0.000000
O	0.000000	0.000000	1.234672

Table S6. Optimized coordinates (Å) of the low spin (LS) CoTPP complex (D_{4h}) at the SR-ZORA, BP86, TZP, spin-unrestricted level of theory.

Co	0.000000	0.000000	0.000000
N	0.000000	1.973938	0.000000
N	0.000000	−1.973938	0.000000
N	1.973938	0.000000	0.000000
N	−1.973938	0.000000	0.000000
C	1.099066	2.825358	0.000000
C	−1.099066	2.825358	0.000000
C	1.099066	−2.825358	0.000000
C	−1.099066	−2.825358	0.000000
C	2.825358	1.099066	0.000000
C	2.825358	−1.099066	0.000000
C	−2.825358	1.099066	0.000000
C	−2.825358	−1.099066	0.000000
C	2.435671	2.435671	0.000000
C	2.435671	−2.435671	0.000000
C	−2.435671	2.435671	0.000000
C	−2.435671	−2.435671	0.000000
C	0.680028	4.202653	0.000000
C	0.680028	−4.202653	0.000000
C	−0.680028	4.202653	0.000000
C	−0.680028	−4.202653	0.000000
C	4.202653	0.680028	0.000000
C	4.202653	−0.680028	0.000000
C	−4.202653	0.680028	0.000000
C	−4.202653	−0.680028	0.000000
H	1.354921	5.052148	0.000000
H	−1.354921	5.052148	0.000000
H	1.354921	−5.052148	0.000000
H	−1.354921	−5.052148	0.000000
H	5.052148	1.354921	0.000000
H	−5.052148	1.354921	0.000000
H	5.052148	−1.354921	0.000000
H	−5.052148	−1.354921	0.000000
C	3.495960	3.495960	0.000000
C	−3.495960	3.495960	0.000000
C	3.495960	−3.495960	0.000000
C	−3.495960	−3.495960	0.000000
C	4.001118	4.001118	1.208092
C	4.001118	4.001118	−1.208092
C	4.001118	−4.001118	1.208092
C	4.001118	−4.001118	−1.208092
C	−4.001118	4.001118	1.208092
C	−4.001118	4.001118	−1.208092
C	−4.001118	−4.001118	1.208092
C	−4.001118	−4.001118	−1.208092
C	4.989395	4.989395	1.208077
C	4.989395	4.989395	−1.208077
C	4.989395	−4.989395	1.208077
C	4.989395	−4.989395	−1.208077
C	−4.989395	4.989395	1.208077
C	−4.989395	4.989395	−1.208077

C	−4.989395	−4.989395	1.208077
C	−4.989395	−4.989395	−1.208077
C	5.486158	5.486158	0.000000
C	−5.486158	5.486158	0.000000
C	5.486158	−5.486158	0.000000
C	−5.486158	−5.486158	0.000000
H	3.613799	3.613799	2.151717
H	3.613799	3.613799	−2.151717
H	3.613799	−3.613799	2.151717
H	3.613799	−3.613799	−2.151717
H	−3.613799	3.613799	2.151717
H	−3.613799	3.613799	−2.151717
H	−3.613799	−3.613799	2.151717
H	−3.613799	−3.613799	−2.151717
H	5.371995	5.371995	2.155576
H	5.371995	5.371995	−2.155576
H	5.371995	−5.371995	2.155576
H	5.371995	−5.371995	−2.155576
H	−5.371995	5.371995	2.155576
H	−5.371995	5.371995	−2.155576
H	−5.371995	−5.371995	2.155576
H	−5.371995	−5.371995	−2.155576
H	6.257766	6.257766	0.000000
H	−6.257766	6.257766	0.000000
H	6.257766	−6.257766	0.000000
H	−6.257766	−6.257766	0.000000

Table S7. Optimized coordinates (Å) of the LS Cu–CoTPP cluster (C_{4v}) at the SR-ZORA, BP86, TZP, spin-restricted level of theory.

Co	0.000000	0.000000	0.180594
N	0.000000	1.964519	0.121918
N	0.000000	−1.964519	0.121918
N	1.964519	0.000000	0.121918
N	−1.964519	0.000000	0.121918
C	1.098522	2.822002	0.109143
C	−1.098522	2.822002	0.109143
C	1.098522	−2.822002	0.109143
C	−1.098522	−2.822002	0.109143
C	2.822002	1.098522	0.109143
C	2.822002	−1.098522	0.109143
C	−2.822002	1.098522	0.109143
C	−2.822002	−1.098522	0.109143
C	2.434235	2.434235	0.098499
C	2.434235	−2.434235	0.098499
C	−2.434235	2.434235	0.098499
C	−2.434235	−2.434235	0.098499
C	0.679895	4.198913	0.087441
C	0.679895	−4.198913	0.087441
C	−0.679895	4.198913	0.087441
C	−0.679895	−4.198913	0.087441
C	4.198913	0.679895	0.087441
C	4.198913	−0.679895	0.087441
C	−4.198913	0.679895	0.087441
C	−4.198913	−0.679895	0.087441
H	1.355836	5.047286	0.069544
H	−1.355836	5.047286	0.069544
H	1.355836	−5.047286	0.069544
H	−1.355836	−5.047286	0.069544
H	5.047286	1.355836	0.069544
H	−5.047286	1.355836	0.069544
H	5.047286	−1.355836	0.069544
H	−5.047286	−1.355836	0.069544
C	3.493487	3.493487	0.041576
C	−3.493487	3.493487	0.041576
C	3.493487	−3.493487	0.041576
C	−3.493487	−3.493487	0.041576
C	4.035429	4.035429	1.216796
C	3.960739	3.960739	−1.196832
C	4.035429	−4.035429	1.216796
C	3.960739	−3.960739	−1.196832
C	−4.035429	4.035429	1.216796
C	−3.960739	3.960739	−1.196832
C	−4.035429	−4.035429	1.216796
C	−3.960739	−3.960739	−1.196832
C	5.022946	5.022946	1.155152
C	4.947837	4.947837	−1.258655
C	5.022946	−5.022946	1.155152
C	4.947837	−4.947837	−1.258655
C	−5.022946	5.022946	1.155152
C	−4.947837	4.947837	−1.258655

C	−5.022946	−5.022946	1.155152
C	−4.947837	−4.947837	−1.258655
C	5.481668	5.481668	−0.082559
C	−5.481668	5.481668	−0.082559
C	5.481668	−5.481668	−0.082559
C	−5.481668	−5.481668	−0.082559
H	3.677553	3.677553	2.183417
H	3.544163	3.544163	−2.115063
H	3.677553	−3.677553	2.183417
H	3.544163	−3.544163	−2.115063
H	−3.677553	3.677553	2.183417
H	−3.544163	3.544163	−2.115063
H	−3.677553	−3.677553	2.183417
H	−3.544163	−3.544163	−2.115063
H	5.434695	5.434695	2.077857
H	5.300196	5.300196	−2.229323
H	5.434695	−5.434695	2.077857
H	5.300196	−5.300196	−2.229323
H	−5.434695	5.434695	2.077857
H	−5.300196	5.300196	−2.229323
H	−5.434695	−5.434695	2.077857
H	−5.300196	−5.300196	−2.229323
H	6.252493	6.252493	−0.130778
H	−6.252493	6.252493	−0.130778
H	6.252493	−6.252493	−0.130778
H	−6.252493	−6.252493	−0.130778
Cu	0.000000	0.000000	2.447107

Table S8. Optimized coordinates (Å) of the LS Ag–CoTPP cluster (C_{4v}) at the SR-ZORA, BP86, TZP, spin-restricted level of theory.

Co	0.000000	0.000000	0.002320
N	0.000000	1.964534	−0.047400
N	0.000000	−1.964534	−0.047400
N	1.964534	0.000000	−0.047400
N	−1.964534	0.000000	−0.047400
C	1.098492	2.822267	−0.044703
C	−1.098492	2.822267	−0.044703
C	1.098492	−2.822267	−0.044703
C	−1.098492	−2.822267	−0.044703
C	2.822267	1.098492	−0.044703
C	2.822267	−1.098492	−0.044703
C	−2.822267	1.098492	−0.044703
C	−2.822267	−1.098492	−0.044703
C	2.434004	2.434004	−0.035185
C	2.434004	−2.434004	−0.035185
C	−2.434004	2.434004	−0.035185
C	−2.434004	−2.434004	−0.035185
C	0.680071	4.199328	−0.053687
C	0.680071	−4.199328	−0.053687
C	−0.680071	4.199328	−0.053687
C	−0.680071	−4.199328	−0.053687
C	4.199328	0.680071	−0.053687
C	4.199328	−0.680071	−0.053687
C	−4.199328	0.680071	−0.053687
C	−4.199328	−0.680071	−0.053687
H	1.356244	5.047614	−0.063645
H	−1.356244	5.047614	−0.063645
H	1.356244	−5.047614	−0.063645
H	−1.356244	−5.047614	−0.063645
H	5.047614	1.356244	−0.063645
H	−5.047614	1.356244	−0.063645
H	5.047614	−1.356244	−0.063645
H	−5.047614	−1.356244	−0.063645
C	3.493510	3.493510	0.005797
C	−3.493510	3.493510	0.005797
C	3.493510	−3.493510	0.005797
C	−3.493510	−3.493510	0.005797
C	3.970259	3.970259	1.236953
C	4.026568	4.026568	−1.177521
C	3.970259	−3.970259	1.236953
C	4.026568	−4.026568	−1.177521
C	−3.970259	3.970259	1.236953
C	−4.026568	4.026568	−1.177521
C	−3.970259	−3.970259	1.236953
C	−4.026568	−4.026568	−1.177521
C	4.957777	4.957777	1.284560
C	5.014375	5.014375	−1.130628
C	4.957777	−4.957777	1.284560
C	5.014375	−5.014375	−1.130628
C	−4.957777	4.957777	1.284560
C	−5.014375	5.014375	−1.130628

C	−4.957777	−4.957777	1.284560
C	−5.014375	−5.014375	−1.130628
C	5.482483	5.482483	0.100193
C	−5.482483	5.482483	0.100193
C	5.482483	−5.482483	0.100193
C	−5.482483	−5.482483	0.100193
H	3.560751	3.560751	2.161570
H	3.661322	3.661322	−2.138468
H	3.560751	−3.560751	2.161570
H	3.661322	−3.661322	−2.138468
H	−3.560751	3.560751	2.161570
H	−3.661322	3.661322	−2.138468
H	−3.560751	−3.560751	2.161570
H	−3.661322	−3.661322	−2.138468
H	5.317665	5.317665	2.249904
H	5.419117	5.419117	−2.059654
H	5.317665	−5.317665	2.249904
H	5.419117	−5.419117	−2.059654
H	−5.317665	5.317665	2.249904
H	−5.419117	5.419117	−2.059654
H	−5.317665	−5.317665	2.249904
H	−5.419117	−5.419117	−2.059654
H	6.253457	6.253457	0.136222
H	−6.253457	6.253457	0.136222
H	6.253457	−6.253457	0.136222
H	−6.253457	−6.253457	0.136222
Ag	0.000000	0.000000	2.475936

Table S9. Optimized coordinates (Å) of the LS Au–CoTPP cluster (C_{4v}) at the SR-ZORA, BP86, TZP, spin-restricted level of theory.

Co	0.000000	0.000000	0.243247
N	0.000000	1.966163	0.145724
N	0.000000	−1.966163	0.145724
N	1.966163	0.000000	0.145724
N	−1.966163	0.000000	0.145724
C	1.098162	2.821823	0.135009
C	−1.098162	2.821823	0.135009
C	1.098162	−2.821823	0.135009
C	−1.098162	−2.821823	0.135009
C	2.821823	1.098162	0.135009
C	2.821823	−1.098162	0.135009
C	−2.821823	1.098162	0.135009
C	−2.821823	−1.098162	0.135009
C	2.433913	2.433913	0.126528
C	2.433913	−2.433913	0.126528
C	−2.433913	2.433913	0.126528
C	−2.433913	−2.433913	0.126528
C	0.679802	4.198671	0.110959
C	0.679802	−4.198671	0.110959
C	−0.679802	4.198671	0.110959
C	−0.679802	−4.198671	0.110959
C	4.198671	0.679802	0.110959
C	4.198671	−0.679802	0.110959
C	−4.198671	0.679802	0.110959
C	−4.198671	−0.679802	0.110959
H	1.356244	5.046495	0.092331
H	−1.356244	5.046495	0.092331
H	1.356244	−5.046495	0.092331
H	−1.356244	−5.046495	0.092331
H	5.046495	1.356244	0.092331
H	−5.046495	1.356244	0.092331
H	5.046495	−1.356244	0.092331
H	−5.046495	−1.356244	0.092331
C	3.493386	3.493386	0.076474
C	−3.493386	3.493386	0.076474
C	3.493386	−3.493386	0.076474
C	−3.493386	−3.493386	0.076474
C	4.030925	4.030925	1.255736
C	3.964328	3.964328	−1.159238
C	4.030925	−4.030925	1.255736
C	3.964328	−3.964328	−1.159238
C	−4.030925	4.030925	1.255736
C	−3.964328	3.964328	−1.159238
C	−4.030925	−4.030925	1.255736
C	−3.964328	−3.964328	−1.159238
C	5.018667	5.018667	1.199918
C	4.951624	4.951624	−1.215358
C	5.018667	−5.018667	1.199918
C	4.951624	−4.951624	−1.215358
C	−5.018667	5.018667	1.199918
C	−4.951624	4.951624	−1.215358

C	−5.018667	−5.018667	1.199918
C	−4.951624	−4.951624	−1.215358
C	5.481671	5.481671	−0.034906
C	−5.481671	5.481671	−0.034906
C	5.481671	−5.481671	−0.034906
C	−5.481671	−5.481671	−0.034906
H	3.669964	3.669964	2.219898
H	3.550810	3.550810	−2.080223
H	3.669964	−3.669964	2.219898
H	3.550810	−3.550810	−2.080223
H	−3.669964	3.669964	2.219898
H	−3.550810	3.550810	−2.080223
H	−3.669964	−3.669964	2.219898
H	−3.550810	−3.550810	−2.080223
H	5.427247	5.427247	2.125554
H	5.306988	5.306988	−2.184010
H	5.427247	−5.427247	2.125554
H	5.306988	−5.306988	−2.184010
H	−5.427247	5.427247	2.125554
H	−5.306988	5.306988	−2.184010
H	−5.427247	−5.427247	2.125554
H	−5.306988	−5.306988	−2.184010
H	6.252528	6.252528	−0.077784
H	−6.252528	6.252528	−0.077784
H	6.252528	−6.252528	−0.077784
H	−6.252528	−6.252528	−0.077784
Au	0.000000	0.000000	2.701329

Table S10. Optimized coordinates (Å) of the LS CoTPP–CO adduct (C_{4v}) at the SR-ZORA, BP86, TZP, spin-unrestricted level of theory.

Co	0.000000	0.000000	−0.247685
N	0.000000	1.987955	−0.002655
N	0.000000	−1.987955	−0.002655
N	1.987955	0.000000	−0.002655
N	−1.987955	0.000000	−0.002655
C	1.099155	2.831102	0.022452
C	−1.099155	2.831102	0.022452
C	1.099155	−2.831102	0.022452
C	−1.099155	−2.831102	0.022452
C	2.831102	1.099155	0.022452
C	2.831102	−1.099155	0.022452
C	−2.831102	1.099155	0.022452
C	−2.831102	−1.099155	0.022452
C	2.437014	2.437014	0.013469
C	2.437014	−2.437014	0.013469
C	−2.437014	2.437014	0.013469
C	−2.437014	−2.437014	0.013469
C	0.680419	4.209418	0.075340
C	0.680419	−4.209418	0.075340
C	−0.680419	4.209418	0.075340
C	−0.680419	−4.209418	0.075340
C	4.209418	0.680419	0.075340
C	4.209418	−0.680419	0.075340
C	−4.209418	0.680419	0.075340
C	−4.209418	−0.680419	0.075340
H	1.355816	5.057602	0.116089
H	−1.355816	5.057602	0.116089
H	1.355816	−5.057602	0.116089
H	−1.355816	−5.057602	0.116089
H	5.057602	1.355816	0.116089
H	−5.057602	1.355816	0.116089
H	5.057602	−1.355816	0.116089
H	−5.057602	−1.355816	0.116089
C	3.496933	3.496933	0.008338
C	−3.496933	3.496933	0.008338
C	3.496933	−3.496933	0.008338
C	−3.496933	−3.496933	0.008338
C	4.008409	4.008409	1.210807
C	3.994644	3.994644	−1.205922
C	4.008409	−4.008409	1.210807
C	3.994644	−3.994644	−1.205922
C	−4.008409	4.008409	1.210807
C	−3.994644	3.994644	−1.205922
C	−4.008409	−4.008409	1.210807
C	−3.994644	−3.994644	−1.205922
C	4.996636	4.996636	1.199116
C	4.982777	4.982777	−1.217624
C	4.996636	−4.996636	1.199116
C	4.982777	−4.982777	−1.217624
C	−4.996636	4.996636	1.199116
C	−4.982777	4.982777	−1.217624

C	−4.996636	−4.996636	1.199116
C	−4.982777	−4.982777	−1.217624
C	5.486237	5.486237	−0.014999
C	−5.486237	5.486237	−0.014999
C	5.486237	−5.486237	−0.014999
C	−5.486237	−5.486237	−0.014999
H	3.626068	3.626068	2.158607
H	3.601610	3.601610	−2.144785
H	3.626068	−3.626068	2.158607
H	3.601610	−3.601610	−2.144785
H	−3.626068	3.626068	2.158607
H	−3.601610	3.601610	−2.144785
H	−3.626068	−3.626068	2.158607
H	−3.601610	−3.601610	−2.144785
H	5.384669	5.384669	2.142154
H	5.359718	5.359718	−2.169659
H	5.384669	−5.384669	2.142154
H	5.359718	−5.359718	−2.169659
H	−5.384669	5.384669	2.142154
H	−5.359718	5.359718	−2.169659
H	−5.384669	−5.384669	2.142154
H	−5.359718	−5.359718	−2.169659
H	6.257750	6.257750	−0.023904
H	−6.257750	6.257750	−0.023904
H	6.257750	−6.257750	−0.023904
H	−6.257750	−6.257750	−0.023904
C	0.000000	0.000000	−2.106731
O	0.000000	0.000000	−3.263621

Table S11. Optimized coordinates (Å) of the LS CoTPP–NH_3 adduct (C_s) at the SR-ZORA, BP86, TZP, spin-unrestricted level of theory.

Co	−0.178880	0.003912	0.000000
N	−0.081012	0.001503	1.982543
N	−0.063172	1.983512	0.000000
N	−0.084230	−1.980362	0.000000
N	−0.081012	0.001503	−1.982543
C	0.112428	−5.495618	5.477516
C	1.287776	−5.023271	4.886077
C	1.230076	−4.033086	3.901757
C	−0.002555	−3.498068	3.494832
C	−1.176288	−3.978502	4.095734
C	−1.120098	−4.970566	5.079120
C	−0.054547	−2.436344	2.437099
C	−0.053499	−1.098360	2.831295
C	−0.049933	1.100472	2.831523
C	−0.007854	0.681444	4.210605
C	−0.008247	−0.679362	4.210364
C	−0.035001	2.438184	2.437398
C	0.000606	3.497743	3.497480
C	1.224142	4.018964	3.947195
C	1.258057	5.007687	4.934019
C	0.068471	5.490336	5.486453
C	−1.155117	4.978966	5.044988
C	−1.187850	3.989081	4.058881
C	−0.027913	2.832074	−1.099525
C	0.026504	4.210480	−0.680453
C	0.026504	4.210480	0.680453
C	−0.027913	2.832074	1.099525
C	−0.035001	2.438184	−2.437398
C	0.000606	3.497743	−3.497480
C	1.224142	4.018964	−3.947195
C	1.258057	5.007687	−4.934019
C	0.068471	5.490336	−5.486453
C	−1.155117	4.978966	−5.044988
C	−1.187850	3.989081	−4.058881
C	−0.067052	−2.829695	−1.098968
C	−0.051275	−4.209564	−0.680261
C	−0.051275	−4.209564	0.680261
C	−0.067052	−2.829695	1.098968
C	−0.054547	−2.436344	−2.437099
C	−0.002555	−3.498068	−3.494832
C	1.230076	−4.033086	−3.901757
C	1.287776	−5.023271	−4.886077
C	0.112428	−5.495618	−5.477516
C	−1.120098	−4.970566	−5.079120
C	−1.176288	−3.978502	−4.095734
C	−0.049933	1.100472	−2.831523
C	−0.007854	0.681444	−4.210605
C	−0.008247	−0.679362	−4.210364
C	−0.053499	−1.098360	−2.831295
H	0.065330	5.059337	−1.355207
H	0.065330	5.059337	1.355207

H	−0.036852	−5.059173	−1.355008
H	−0.036852	−5.059173	1.355008
H	0.031080	1.355784	−5.059857
H	0.031080	1.355784	5.059857
H	0.030694	−1.354137	−5.059197
H	0.030694	−1.354137	5.059197
H	2.153516	3.642257	−3.517327
H	−2.144090	3.590646	−3.715974
H	2.147977	−3.664775	−3.440940
H	−2.139894	−3.571129	−3.785361
H	2.153516	3.642257	3.517327
H	−2.144090	3.590646	3.715974
H	2.147977	−3.664775	3.440940
H	−2.139894	−3.571129	3.785361
H	2.217996	5.400365	−5.273599
H	−2.088967	5.352900	−5.468235
H	2.255020	−5.425333	−5.192462
H	−2.042483	−5.334877	−5.534320
H	2.217996	5.400365	5.273599
H	−2.088967	5.352900	5.468235
H	2.255020	−5.425333	5.192462
H	−2.042483	−5.334877	5.534320
H	0.094858	6.262180	−6.257564
H	0.094858	6.262180	6.257564
H	0.156967	−6.269042	−6.246077
H	0.156967	−6.269042	6.246077
N	−2.373157	−0.006434	0.000000
H	−2.748922	0.944682	0.000000
H	−2.737225	−0.486201	−0.826526
H	−2.737225	−0.486201	0.826526

Table S12. Optimized coordinates (Å) of the LS CoTPP–NO cluster (C_s) at the SR-ZORA, BP86, TZP, spin-restricted level of theory.

Co	−0.083172	0.061058	0.000000
N	1.065793	0.891712	1.382307
N	−0.943760	−1.030220	−1.382943
N	1.065793	0.891712	−1.382307
N	−0.943760	−1.030220	1.382943
C	1.859300	2.013871	1.220692
C	1.133120	0.584934	2.730341
C	−0.636875	−1.103163	−2.732801
C	−2.074976	−1.813095	−1.221115
C	1.859300	2.013871	−1.220692
C	1.133120	0.584934	−2.730341
C	−0.636875	−1.103163	2.732801
C	−2.074976	−1.813095	1.221115
C	2.176476	2.613506	0.000000
C	0.375938	−0.391802	−3.379421
C	0.375938	−0.391802	3.379421
C	−2.671308	−2.133356	0.000000
C	2.425040	2.414615	2.484632
C	−1.572449	−1.964834	−3.411197
C	2.005918	1.508465	3.411533
C	−2.484325	−2.371475	−2.484919
C	2.425040	2.414615	−2.484632
C	2.005918	1.508465	−3.411533
C	−1.572449	−1.964834	3.411197
C	−2.484325	−2.371475	2.484919
H	3.083948	3.265235	2.623965
H	2.236669	1.473185	4.471062
H	−1.535255	−2.205135	−4.468478
H	−3.340895	−3.022644	−2.623736
H	3.083948	3.265235	−2.623965
H	−1.535255	−2.205135	4.468478
H	2.236669	1.473185	−4.471062
H	−3.340895	−3.022644	2.623736
C	2.975858	3.879601	0.000000
C	0.606242	−0.624841	4.840014
C	0.606242	−0.624841	−4.840014
C	−3.923196	−2.955766	0.000000
C	4.379256	3.856964	0.000000
C	2.317664	5.119108	0.000000
C	1.666002	−1.440864	−5.265187
C	−0.222386	−0.032344	−5.805588
C	1.666002	−1.440864	5.265187
C	−0.222386	−0.032344	5.805588
C	−3.865028	−4.358212	0.000000
C	−5.178688	−2.328723	0.000000
C	5.108513	5.048947	0.000000
C	3.047034	6.310926	0.000000
C	1.890091	−1.663083	−6.626648
C	0.002844	−0.252646	−7.166661
C	1.890091	−1.663083	6.626648
C	0.002844	−0.252646	7.166661

C	−5.037837	−5.117659	0.000000
C	−6.351535	−3.088770	0.000000
C	4.444127	6.278770	0.000000
C	1.058924	−1.069748	7.580651
C	1.058924	−1.069748	−7.580651
C	−6.283976	−4.484603	0.000000
H	4.898135	2.897150	0.000000
H	1.227057	5.143127	0.000000
H	2.314170	−1.904553	−4.520229
H	−1.045133	0.606926	−5.482328
H	2.314170	−1.904553	4.520229
H	−1.045133	0.606926	5.482328
H	−2.892404	−4.852611	0.000000
H	−5.230062	−1.239142	0.000000
H	6.199133	5.015771	0.000000
H	2.520689	7.266561	0.000000
H	2.716220	−2.302482	−6.941675
H	−0.648261	0.217612	−7.905312
H	2.716220	−2.302482	6.941675
H	−0.648261	0.217612	7.905312
H	−4.976911	−6.207138	0.000000
H	−7.320529	−2.587472	0.000000
H	5.013991	7.209074	0.000000
H	1.233978	−1.242818	8.643576
H	1.233978	−1.242818	−8.643576
H	−7.199499	−5.077865	0.000000
N	−1.288775	1.404477	0.000000
O	−2.458227	1.211738	0.000000

Table S13. Optimized coordinates (Å) of the LS CoTPP–NO_2 cluster (C_{2v}) at the SR-ZORA, BP86, TZP, spin-restricted level of theory.

Co	0.000000	0.000000	−0.225771
N	1.385389	1.385097	−0.022055
N	−1.385389	−1.385097	−0.022055
N	1.385389	−1.385097	−0.022055
N	−1.385389	1.385097	−0.022055
C	2.749686	1.219553	−0.208416
C	1.219357	2.743951	0.209720
C	−1.219357	−2.743951	0.209720
C	−2.749686	−1.219553	−0.208416
C	2.749686	−1.219553	−0.208416
C	1.219357	−2.743951	0.209720
C	−1.219357	2.743951	0.209720
C	−2.749686	1.219553	−0.208416
C	3.405937	0.000000	−0.363504
C	0.000000	−3.399136	0.370510
C	0.000000	3.399136	0.370510
C	−3.405937	0.000000	−0.363504
C	3.434024	2.481380	−0.098339
C	−2.491040	−3.419177	0.192617
C	2.491040	3.419177	0.192617
C	−3.434024	−2.481380	−0.098339
C	3.434024	−2.481380	−0.098339
C	2.491040	−3.419177	0.192617
C	−2.491040	3.419177	0.192617
C	−3.434024	2.481380	−0.098339
H	4.506022	2.611038	−0.202829
H	2.625669	4.481558	0.366465
H	−2.625669	−4.481558	0.366465
H	−4.506022	−2.611038	−0.202829
H	4.506022	−2.611038	−0.202829
H	−2.625669	4.481558	0.366465
H	2.625669	−4.481558	0.366465
H	−4.506022	2.611038	−0.202829
C	4.883706	0.000000	−0.606050
C	0.000000	4.867198	0.668882
C	0.000000	−4.867198	0.668882
C	−4.883706	0.000000	−0.606050
C	5.790965	0.000000	0.464753
C	5.379321	0.000000	−1.918843
C	0.000000	−5.315528	1.998740
C	0.000000	−5.814123	−0.366837
C	0.000000	5.315528	1.998740
C	0.000000	5.814123	−0.366837
C	−5.790965	0.000000	0.464753
C	−5.379321	0.000000	−1.918843
C	7.167966	0.000000	0.227719
C	6.756907	0.000000	−2.154499
C	0.000000	−6.683141	2.286924
C	0.000000	−7.181339	−0.078053
C	0.000000	6.683141	2.286924
C	0.000000	7.181339	−0.078053

C	−7.167966	0.000000	0.227719
C	−6.756907	0.000000	−2.154499
C	7.653991	0.000000	−1.082770
C	0.000000	7.619180	1.249343
C	0.000000	−7.619180	1.249343
C	−7.653991	0.000000	−1.082770
H	5.411047	0.000000	1.487539
H	4.677059	0.000000	−2.753406
H	0.000000	−4.585442	2.809402
H	0.000000	−5.472073	−1.402948
H	0.000000	4.585442	2.809402
H	0.000000	5.472073	−1.402948
H	−5.411047	0.000000	1.487539
H	−4.677059	0.000000	−2.753406
H	7.861846	0.000000	1.069776
H	7.129311	0.000000	−3.179946
H	0.000000	−7.016842	3.325690
H	0.000000	−7.905702	−0.893883
H	0.000000	7.016842	3.325690
H	0.000000	7.905702	−0.893883
H	−7.861846	0.000000	1.069776
H	−7.129311	0.000000	−3.179946
H	8.729114	0.000000	−1.267908
H	0.000000	8.686670	1.474354
H	0.000000	−8.686670	1.474354
H	−8.729114	0.000000	−1.267908
N	0.000000	0.000000	−2.107458
O	−1.096721	0.000000	−2.674464
O	1.096721	0.000000	−2.674464

Table S14. Optimized coordinates (Å) of the LS CoTPP–O_2 cluster (C_s) at the SR-ZORA, BP86, TZP, spin-unrestricted level of theory.

Co	−0.093828	0.074786	0.000000
N	1.015598	0.934233	1.373235
N	−0.938511	−1.024578	−1.375058
N	1.015598	0.934233	−1.373235
N	−0.938511	−1.024578	1.375058
C	1.778500	2.081116	1.219257
C	1.114101	0.607316	2.716149
C	−0.608863	−1.123314	−2.717257
C	−2.080445	−1.790660	−1.220022
C	1.778500	2.081116	−1.219257
C	1.114101	0.607316	−2.716149
C	−0.608863	−1.123314	2.717257
C	−2.080445	−1.790660	1.220022
C	2.086353	2.687837	0.000000
C	0.404923	−0.409591	−3.357896
C	0.404923	−0.409591	3.357896
C	−2.688361	−2.093357	0.000000
C	2.346218	2.480121	2.482116
C	−1.538416	−1.990264	−3.396976
C	1.969651	1.545182	3.398789
C	−2.474410	−2.365861	−2.481224
C	2.346218	2.480121	−2.482116
C	1.969651	1.545182	−3.398789
C	−1.538416	−1.990264	3.396976
C	−2.474410	−2.365861	2.481224
H	2.980473	3.348613	2.626691
H	2.218612	1.498299	4.453929
H	−1.484886	−2.247691	−4.449691
H	−3.338399	−3.006318	−2.624993
H	2.980473	3.348613	−2.626691
H	−1.484886	−2.247691	4.449691
H	2.218612	1.498299	−4.453929
H	−3.338399	−3.006318	2.624993
C	2.894022	3.949162	0.000000
C	0.684295	−0.689670	4.802031
C	0.684295	−0.689670	−4.802031
C	−3.957956	−2.885812	0.000000
C	4.297169	3.905317	0.000000
C	2.254160	5.198120	0.000000
C	1.721963	−1.565728	−5.155428
C	−0.080846	−0.098468	−5.819522
C	1.721963	−1.565728	5.155428
C	−0.080846	−0.098468	5.819522
C	−3.935159	−4.289115	0.000000
C	−5.195640	−2.224725	0.000000
C	5.044845	5.085684	0.000000
C	3.002627	6.378467	0.000000
C	1.985039	−1.850742	−6.497822
C	0.183403	−0.382645	−7.161984
C	1.985039	−1.850742	6.497822
C	0.183403	−0.382645	7.161984

C	−5.127485	−5.017581	0.000000
C	−6.387493	−2.954322	0.000000
C	4.399155	6.325256	0.000000
C	1.215566	−1.261039	7.504451
C	1.215566	−1.261039	−7.504451
C	−6.356368	−4.351669	0.000000
H	4.801652	2.937891	0.000000
H	1.163996	5.239624	0.000000
H	2.320943	−2.028280	−4.369600
H	−0.887604	0.585909	−5.552294
H	2.320943	−2.028280	4.369600
H	−0.887604	0.585909	5.552294
H	−2.975640	−4.808476	0.000000
H	−5.216158	−1.134235	0.000000
H	6.134879	5.035662	0.000000
H	2.491307	7.342148	0.000000
H	2.791477	−2.538782	−6.756557
H	−0.421391	0.082241	−7.942209
H	2.791477	−2.538782	6.756557
H	−0.421391	0.082241	7.942209
H	−5.094937	−6.108256	0.000000
H	−7.342811	−2.427538	0.000000
H	4.983264	7.246681	0.000000
H	1.418081	−1.486788	8.552499
H	1.418081	−1.486788	−8.552499
H	−7.287056	−4.920840	0.000000
O	−1.346154	1.447836	0.000000
O	−2.600547	1.166532	0.000000

Table S15. Optimized coordinates (Å) of the LS Cu–CoTPP–CO cluster (C_{4v}) at the SR-ZORA, BP86, TZP, spin-restricted level of theory.

Co	0.000000	0.000000	−0.023675
N	0.000000	1.997992	0.095059
N	0.000000	−1.997992	0.095059
N	1.997992	0.000000	0.095059
N	−1.997992	0.000000	0.095059
C	1.099986	2.834725	0.090516
C	−1.099986	2.834725	0.090516
C	1.099986	−2.834725	0.090516
C	−1.099986	−2.834725	0.090516
C	2.834725	1.099986	0.090516
C	2.834725	−1.099986	0.090516
C	−2.834725	1.099986	0.090516
C	−2.834725	−1.099986	0.090516
C	2.438600	2.438600	0.074029
C	2.438600	−2.438600	0.074029
C	−2.438600	2.438600	0.074029
C	−2.438600	−2.438600	0.074029
C	0.680426	4.214590	0.093202
C	0.680426	−4.214590	0.093202
C	−0.680426	4.214590	0.093202
C	−0.680426	−4.214590	0.093202
C	4.214590	0.680426	0.093202
C	4.214590	−0.680426	0.093202
C	−4.214590	0.680426	0.093202
C	−4.214590	−0.680426	0.093202
H	1.356220	5.063087	0.095012
H	−1.356220	5.063087	0.095012
H	1.356220	−5.063087	0.095012
H	−1.356220	−5.063087	0.095012
H	5.063087	1.356220	0.095012
H	−5.063087	1.356220	0.095012
H	5.063087	−1.356220	0.095012
H	−5.063087	−1.356220	0.095012
C	3.497269	3.497269	−0.006293
C	−3.497269	3.497269	−0.006293
C	3.497269	−3.497269	−0.006293
C	−3.497269	−3.497269	−0.006293
C	4.056885	4.056885	1.152194
C	3.945709	3.945709	−1.259052
C	4.056885	−4.056885	1.152194
C	3.945709	−3.945709	−1.259052
C	−4.056885	4.056885	1.152194
C	−3.945709	3.945709	−1.259052
C	−4.056885	−4.056885	1.152194
C	−3.945709	−3.945709	−1.259052
C	5.043337	5.043337	1.059462
C	4.931514	4.931514	−1.351766
C	5.043337	−5.043337	1.059462
C	4.931514	−4.931514	−1.351766
C	−5.043337	5.043337	1.059462
C	−4.931514	4.931514	−1.351766

C	−5.043337	−5.043337	1.059462
C	−4.931514	−4.931514	−1.351766
C	5.482921	5.482921	−0.192120
C	−5.482921	5.482921	−0.192120
C	5.482921	−5.482921	−0.192120
C	−5.482921	−5.482921	−0.192120
H	3.713902	3.713902	2.129528
H	3.515133	3.515133	−2.164375
H	3.713902	−3.713902	2.129528
H	3.515133	−3.515133	−2.164375
H	−3.713902	3.713902	2.129528
H	−3.515133	3.515133	−2.164375
H	−3.713902	−3.713902	2.129528
H	−3.515133	−3.515133	−2.164375
H	5.469416	5.469416	1.968959
H	5.268866	5.268866	−2.332996
H	5.469416	−5.469416	1.968959
H	5.268866	−5.268866	−2.332996
H	−5.469416	5.469416	1.968959
H	−5.268866	5.268866	−2.332996
H	−5.469416	−5.469416	1.968959
H	−5.268866	−5.268866	−2.332996
H	6.252700	6.252700	−0.264007
H	−6.252700	6.252700	−0.264007
H	6.252700	−6.252700	−0.264007
H	−6.252700	−6.252700	−0.264007
C	0.000000	0.000000	−1.907196
O	0.000000	0.000000	−3.060782
Cu	0.000000	0.000000	2.319456

Table S16. Optimized coordinates (Å) of the LS Ag–CoTPP–CO cluster (C_{4v}) at the SR-ZORA, BP86, TZP, spin-restricted level of theory.

Co	0.000000	0.000000	−0.197261
N	0.000000	1.999222	−0.070306
N	0.000000	−1.999222	−0.070306
N	1.999222	0.000000	−0.070306
N	−1.999222	0.000000	−0.070306
C	1.100120	2.835858	−0.048948
C	−1.100120	2.835858	−0.048948
C	1.100120	−2.835858	−0.048948
C	−1.100120	−2.835858	−0.048948
C	2.835858	1.100120	−0.048948
C	2.835858	−1.100120	−0.048948
C	−2.835858	1.100120	−0.048948
C	−2.835858	−1.100120	−0.048948
C	2.438694	2.438694	−0.037898
C	2.438694	−2.438694	−0.037898
C	−2.438694	2.438694	−0.037898
C	−2.438694	−2.438694	−0.037898
C	0.680322	4.215902	−0.018950
C	0.680322	−4.215902	−0.018950
C	−0.680322	4.215902	−0.018950
C	−0.680322	−4.215902	−0.018950
C	4.215902	0.680322	−0.018950
C	4.215902	−0.680322	−0.018950
C	−4.215902	0.680322	−0.018950
C	−4.215902	−0.680322	−0.018950
H	1.355308	5.064920	0.003567
H	−1.355308	5.064920	0.003567
H	1.355308	−5.064920	0.003567
H	−1.355308	−5.064920	0.003567
H	5.064920	1.355308	0.003567
H	−5.064920	1.355308	0.003567
H	5.064920	−1.355308	0.003567
H	−5.064920	−1.355308	0.003567
C	3.498324	3.498324	0.013369
C	−3.498324	3.498324	0.013369
C	3.498324	−3.498324	0.013369
C	−3.498324	−3.498324	0.013369
C	3.970843	3.970843	1.247816
C	4.035221	4.035221	−1.166783
C	3.970843	−3.970843	1.247816
C	4.035221	−4.035221	−1.166783
C	−3.970843	3.970843	1.247816
C	−4.035221	4.035221	−1.166783
C	−3.970843	−3.970843	1.247816
C	−4.035221	−4.035221	−1.166783
C	4.958259	4.958259	1.301222
C	5.022827	5.022827	−1.113670
C	4.958259	−4.958259	1.301222
C	5.022827	−5.022827	−1.113670
C	−4.958259	4.958259	1.301222
C	−5.022827	5.022827	−1.113670

C	−4.958259	−4.958259	1.301222
C	−5.022827	−5.022827	−1.113670
C	5.486814	5.486814	0.120280
C	−5.486814	5.486814	0.120280
C	5.486814	−5.486814	0.120280
C	−5.486814	−5.486814	0.120280
H	3.558078	3.558078	2.169515
H	3.673430	3.673430	−2.130371
H	3.558078	−3.558078	2.169515
H	3.673430	−3.673430	−2.130371
H	−3.558078	3.558078	2.169515
H	−3.673430	3.673430	−2.130371
H	−3.558078	−3.558078	2.169515
H	−3.673430	−3.673430	−2.130371
H	5.314882	5.314882	2.268982
H	5.430312	5.430312	−2.040282
H	5.314882	−5.314882	2.268982
H	5.430312	−5.430312	−2.040282
H	−5.314882	5.314882	2.268982
H	−5.430312	5.430312	−2.040282
H	−5.314882	−5.314882	2.268982
H	−5.430312	−5.430312	−2.040282
H	6.257663	6.257663	0.161512
H	−6.257663	6.257663	0.161512
H	6.257663	−6.257663	0.161512
H	−6.257663	−6.257663	0.161512
C	0.000000	0.000000	−2.074707
O	0.000000	0.000000	−3.228478
Ag	0.000000	0.000000	2.371817

Table S17. Optimized coordinates (Å) of the LS Au–CoTPP–CO cluster (C_{4v}) at the SR-ZORA, BP86, TZP, spin-restricted level of theory.

Co	0.000000	0.000000	−0.069002
N	0.000000	2.001384	0.010261
N	0.000000	−2.001384	0.010261
N	2.001384	0.000000	0.010261
N	−2.001384	0.000000	0.010261
C	1.101308	2.835620	0.014768
C	−1.101308	2.835620	0.014768
C	1.101308	−2.835620	0.014768
C	−1.101308	−2.835620	0.014768
C	2.835620	1.101308	0.014768
C	2.835620	−1.101308	0.014768
C	−2.835620	1.101308	0.014768
C	−2.835620	−1.101308	0.014768
C	2.439872	2.439872	0.009524
C	2.439872	−2.439872	0.009524
C	−2.439872	2.439872	0.009524
C	−2.439872	−2.439872	0.009524
C	0.680252	4.215007	0.027799
C	0.680252	−4.215007	0.027799
C	−0.680252	4.215007	0.027799
C	−0.680252	−4.215007	0.027799
C	4.215007	0.680252	0.027799
C	4.215007	−0.680252	0.027799
C	−4.215007	0.680252	0.027799
C	−4.215007	−0.680252	0.027799
H	1.355936	5.063545	0.040910
H	−1.355936	5.063545	0.040910
H	1.355936	−5.063545	0.040910
H	−1.355936	−5.063545	0.040910
H	5.063545	1.355936	0.040910
H	−5.063545	1.355936	0.040910
H	5.063545	−1.355936	0.040910
H	−5.063545	−1.355936	0.040910
C	3.499738	3.499738	−0.027220
C	−3.499738	3.499738	−0.027220
C	3.499738	−3.499738	−0.027220
C	−3.499738	−3.499738	−0.027220
C	4.031351	4.031351	1.157645
C	3.976892	3.976892	−1.258210
C	4.031351	−4.031351	1.157645
C	3.976892	−3.976892	−1.258210
C	−4.031351	4.031351	1.157645
C	−3.976892	3.976892	−1.258210
C	−4.031351	−4.031351	1.157645
C	−3.976892	−3.976892	−1.258210
C	5.019315	5.019315	1.111286
C	4.964395	4.964395	−1.304256
C	5.019315	−5.019315	1.111286
C	4.964395	−4.964395	−1.304256
C	−5.019315	5.019315	1.111286
C	−4.964395	4.964395	−1.304256

C	−5.019315	−5.019315	1.111286
C	−4.964395	−4.964395	−1.304256
C	5.488128	5.488128	−0.119101
C	−5.488128	5.488128	−0.119101
C	5.488128	−5.488128	−0.119101
C	−5.488128	−5.488128	−0.119101
H	3.665172	3.665172	2.117857
H	3.568195	3.568195	−2.183526
H	3.665172	−3.665172	2.117857
H	3.568195	−3.568195	−2.183526
H	−3.665172	3.665172	2.117857
H	−3.568195	3.568195	−2.183526
H	−3.665172	−3.665172	2.117857
H	−3.568195	−3.568195	−2.183526
H	5.423572	5.423572	2.040662
H	5.324555	5.324555	−2.269277
H	5.423572	−5.423572	2.040662
H	5.324555	−5.324555	−2.269277
H	−5.423572	5.423572	2.040662
H	−5.324555	5.324555	−2.269277
H	−5.423572	−5.423572	2.040662
H	−5.324555	−5.324555	−2.269277
H	6.259157	6.259157	−0.154599
H	−6.259157	6.259157	−0.154599
H	6.259157	−6.259157	−0.154599
H	−6.259157	−6.259157	−0.154599
C	0.000000	0.000000	−1.890884
O	0.000000	0.000000	−3.043338
Au	0.000000	0.000000	2.453447

Table S18. Optimized coordinates (Å) of the LS Cu–CoTPP–NH_3 (C_s) cluster at the SR-ZORA, BP86, TZP, spin-restricted level of theory.

Co	−0.029672	0.009156	0.000000
N	−0.002553	0.005436	1.985150
N	0.021361	1.989773	0.000000
N	−0.059649	−1.975289	0.000000
N	−0.002553	0.005436	−1.985150
C	−0.016080	−5.516293	5.454277
C	1.177385	−4.889334	5.084889
C	1.172489	−3.886714	4.111106
C	−0.024989	−3.496057	3.491642
C	−1.217792	−4.131571	3.871013
C	−1.214390	−5.134016	4.845008
C	−0.027615	−2.431568	2.437085
C	0.007007	−1.094223	2.831554
C	0.025744	1.103392	2.832341
C	0.056385	0.683663	4.211255
C	0.039295	−0.676613	4.210705
C	0.024301	2.441860	2.437955
C	0.022199	3.501023	3.495202
C	1.191105	4.219255	3.791995
C	1.187033	5.211848	4.775121
C	0.012409	5.502385	5.475047
C	−1.157158	4.792529	5.187872
C	−1.151366	3.799169	4.205446
C	0.015919	2.836199	−1.099283
C	−0.018135	4.215001	−0.680076
C	−0.018135	4.215001	0.680076
C	0.015919	2.836199	1.099283
C	0.024301	2.441860	−2.437955
C	0.022199	3.501023	−3.495202
C	1.191105	4.219255	−3.791995
C	1.187033	5.211848	−4.775121
C	0.012409	5.502385	−5.475047
C	−1.157158	4.792529	−5.187872
C	−1.151366	3.799169	−4.205446
C	−0.060580	−2.822491	−1.098466
C	−0.070525	−4.202020	−0.679891
C	−0.070525	−4.202020	0.679891
C	−0.060580	−2.822491	1.098466
C	−0.027615	−2.431568	−2.437085
C	−0.024989	−3.496057	−3.491642
C	1.172489	−3.886714	−4.111106
C	1.177385	−4.889334	−5.084889
C	−0.016080	−5.516293	−5.454277
C	−1.214390	−5.134016	−4.845008
C	−1.217792	−4.131571	−3.871013
C	0.025744	1.103392	−2.832341
C	0.056385	0.683663	−4.211255
C	0.039295	−0.676613	−4.210705
C	0.007007	−1.094223	−2.831554
H	−0.046302	5.064751	−1.353497
H	−0.046302	5.064751	1.353497

H	−0.066841	−5.052068	−1.353725
H	−0.066841	−5.052068	1.353725
H	0.086863	1.357153	−5.061318
H	0.086863	1.357153	5.061318
H	0.048408	−1.351740	−5.060001
H	0.048408	−1.351740	5.060001
H	2.107744	3.990977	−3.246868
H	−2.064825	3.247059	−3.980412
H	2.105090	−3.402297	−3.818497
H	−2.153982	−3.835322	−3.395352
H	2.107744	3.990977	3.246868
H	−2.064825	3.247059	3.980412
H	2.105090	−3.402297	3.818497
H	−2.153982	−3.835322	3.395352
H	2.105122	5.758461	−4.995721
H	−2.078785	5.014822	−5.728476
H	2.117897	−5.184970	−5.552231
H	−2.150522	−5.617601	−5.128642
H	2.105122	5.758461	4.995721
H	−2.078785	5.014822	5.728476
H	2.117897	−5.184970	5.552231
H	−2.150522	−5.617601	5.128642
H	0.008192	6.278805	−6.241594
H	0.008192	6.278805	6.241594
H	−0.012247	−6.301492	−6.212406
H	−0.012247	−6.301492	6.212406
N	−2.227677	0.044069	0.000000
H	−2.578794	1.005294	0.000000
H	−2.602000	−0.426949	−0.827541
H	−2.602000	−0.426949	0.827541
Cu	2.274756	−0.035064	0.000000

Table S19. Optimized coordinates (Å) of the LS Ag–CoTPP–NH_3 (C_s) cluster at the SR-ZORA, BP86, TZP, spin-restricted level of theory.

Co	−0.025312	0.006710	0.000000
N	0.019389	0.004183	1.983379
N	−0.020258	1.986635	0.000000
N	−0.026403	−1.976246	0.000000
N	0.019389	0.004183	−1.983379
C	0.051563	−5.497938	5.473321
C	1.245684	−5.085658	4.874479
C	1.231850	−4.093320	3.890463
C	0.025483	−3.494964	3.493799
C	−1.167097	−3.914074	4.104007
C	−1.155492	−4.910632	5.084170
C	0.011461	−2.432393	2.436105
C	0.062076	−1.095184	2.829320
C	0.066220	1.102587	2.829572
C	0.151320	0.683566	4.206667
C	0.149162	−0.676915	4.206262
C	0.018579	2.440119	2.436183
C	0.034463	3.497888	3.497661
C	1.234340	4.130757	3.857639
C	1.252126	5.110314	4.854340
C	0.069112	5.472478	5.504499
C	−1.131890	4.850213	5.152222
C	−1.147654	3.868463	4.157649
C	−0.041293	2.833204	−1.099073
C	−0.110082	4.211400	−0.680206
C	−0.110082	4.211400	0.680206
C	−0.041293	2.833204	1.099073
C	0.018579	2.440119	−2.436183
C	0.034463	3.497888	−3.497661
C	1.234340	4.130757	−3.857639
C	1.252126	5.110314	−4.854340
C	0.069112	5.472478	−5.504499
C	−1.131890	4.850213	−5.152222
C	−1.147654	3.868463	−4.157649
C	−0.046530	−2.823677	−1.098541
C	−0.111239	−4.202155	−0.680186
C	−0.111239	−4.202155	0.680186
C	−0.046530	−2.823677	1.098541
C	0.011461	−2.432393	−2.436105
C	0.025483	−3.494964	−3.493799
C	1.231850	−4.093320	−3.890463
C	1.245684	−5.085658	−4.874479
C	0.051563	−5.497938	−5.473321
C	−1.155492	−4.910632	−5.084170
C	−1.167097	−3.914074	−4.104007
C	0.066220	1.102587	−2.829572
C	0.151320	0.683566	−4.206667
C	0.149162	−0.676915	−4.206262
C	0.062076	−1.095184	−2.829320
H	−0.147646	5.060096	−1.355428
H	−0.147646	5.060096	1.355428

H	−0.145722	−5.050796	−1.355572
H	−0.145722	−5.050796	1.355572
H	0.206732	1.357929	−5.054652
H	0.206732	1.357929	5.054652
H	0.201501	−1.352517	−5.053731
H	0.201501	−1.352517	5.053731
H	2.158138	3.845048	−3.352261
H	−2.085357	3.381663	−3.884612
H	2.163927	−3.771562	−3.423435
H	−2.110795	−3.457252	−3.801046
H	2.158138	3.845048	3.352261
H	−2.085357	3.381663	3.884612
H	2.163927	−3.771562	3.423435
H	−2.110795	−3.457252	3.801046
H	2.194186	5.588911	−5.126484
H	−2.060108	5.129153	−5.653595
H	2.192536	−5.537728	−5.174367
H	−2.092634	−5.228608	−5.544509
H	2.194186	5.588911	5.126484
H	−2.060108	5.129153	5.653595
H	2.192536	−5.537728	5.174367
H	−2.092634	−5.228608	5.544509
H	0.082668	6.237245	−6.282508
H	0.082668	6.237245	6.282508
H	0.061682	−6.274481	−6.239561
H	0.061682	−6.274481	6.239561
N	−2.233473	0.000936	0.000000
H	−2.603190	0.955210	0.000000
H	−2.597997	−0.477765	−0.827385
H	−2.597997	−0.477765	0.827385
Ag	2.492203	0.000923	0.000000

Table S20. Optimized coordinates (Å) of the LS Au–CoTPP–NH_3 (C_s) cluster at the SR-ZORA, BP86, TZP, spin-restricted level of theory.

Co	−0.012744	0.010599	0.000000
N	−0.002107	0.006870	−1.989800
N	−0.002107	0.006870	1.989800
N	0.019791	1.994432	0.000000
N	−0.046701	−1.980148	0.000000
C	0.171788	−5.463904	5.500194
C	1.347595	−4.991591	4.909871
C	1.290300	−4.012367	3.914635
C	0.056164	−3.490059	3.496112
C	−1.118347	−3.971356	4.093992
C	−1.061890	−4.952125	5.088464
C	0.004548	−2.431357	2.436053
C	0.034259	−1.093962	2.830512
C	0.047696	1.104453	2.833096
C	0.114065	0.683126	4.210504
C	0.105527	−0.677357	4.208820
C	0.044399	2.442635	2.438426
C	0.073711	3.502598	3.495156
C	1.260006	4.198308	3.777000
C	1.285064	5.191774	4.759236
C	0.125191	5.503975	5.473447
C	−1.060860	4.816608	5.201261
C	−1.085307	3.822665	4.219532
C	0.021458	2.836731	−1.099787
C	−0.008361	4.216163	−0.680089
C	−0.008361	4.216163	0.680089
C	0.021458	2.836731	1.099787
C	0.044399	2.442635	−2.438426
C	0.073711	3.502598	−3.495156
C	1.260006	4.198308	−3.777000
C	1.285064	5.191774	−4.759236
C	0.125191	5.503975	−5.473447
C	−1.060860	4.816608	−5.201261
C	−1.085307	3.822665	−4.219532
C	−0.046550	−2.823812	−1.098527
C	−0.077662	−4.203582	−0.680064
C	−0.077662	−4.203582	0.680064
C	−0.046550	−2.823812	1.098527
C	0.004548	−2.431357	−2.436053
C	0.056164	−3.490059	−3.496112
C	1.290300	−4.012367	−3.914635
C	1.347595	−4.991591	−4.909871
C	0.171788	−5.463904	−5.500194
C	−1.061890	−4.952125	−5.088464
C	−1.118347	−3.971356	−4.093992
C	0.047696	1.104453	−2.833096
C	0.114065	0.683126	−4.210504
C	0.105527	−0.677357	−4.208820
C	0.034259	−1.093962	−2.830512
H	−0.030001	5.065822	−1.354189
H	−0.030001	5.065822	1.354189

H	−0.088339	−5.052159	−1.356393
H	−0.088339	−5.052159	1.356393
H	0.167787	1.355517	−5.059759
H	0.167787	1.355517	5.059759
H	0.148288	−1.353295	−5.056319
H	0.148288	−1.353295	5.056319
H	2.165518	3.951856	−3.221125
H	−2.012055	3.287997	−4.005859
H	2.207417	−3.642807	−3.453762
H	−2.082636	−3.571345	−3.775652
H	2.165518	3.951856	3.221125
H	−2.012055	3.287997	4.005859
H	2.207417	−3.642807	3.453762
H	−2.082636	−3.571345	3.775652
H	2.215208	5.722180	−4.968510
H	−1.971430	5.055307	−5.753300
H	2.314752	−5.386573	−5.225311
H	−1.984722	−5.316892	−5.542912
H	2.215208	5.722180	4.968510
H	−1.971430	5.055307	5.753300
H	2.314752	−5.386573	5.225311
H	−1.984722	−5.316892	5.542912
H	0.145777	6.280054	−6.240104
H	0.145777	6.280054	6.240104
H	0.216881	−6.228637	−6.277066
H	0.216881	−6.228637	6.277066
N	−2.096528	0.038335	0.000000
H	−2.439256	1.003000	0.000000
H	−2.462055	−0.434161	−0.830782
H	−2.462055	−0.434161	0.830782
Au	2.484464	−0.035576	0.000000

Table S21. Optimized coordinates (Å) of the LS Cu–CoTPP–NO (C_s) cluster at the SR-ZORA, BP86, TZP, spin-unrestricted level of theory.

Co	0.047100	−0.048040	0.000000
N	1.126926	0.852278	1.398941
N	−0.848054	−1.117647	−1.395579
N	1.126926	0.852278	−1.398941
N	−0.848054	−1.117647	1.395579
C	1.937107	1.960365	1.224447
C	1.137268	0.600579	2.761520
C	−0.595846	−1.127251	−2.758567
C	−1.956755	−1.929576	−1.223311
C	1.937107	1.960365	−1.224447
C	1.137268	0.600579	−2.761520
C	−0.595846	−1.127251	2.758567
C	−1.956755	−1.929576	1.223311
C	2.273480	2.541819	0.000000
C	0.359752	−0.353577	−3.419908
C	0.359752	−0.353577	3.419908
C	−2.535406	−2.272575	0.000000
C	2.464065	2.401505	2.491778
C	−1.540659	−1.978635	−3.435801
C	1.993517	1.542475	3.437708
C	−2.399324	−2.451781	−2.491118
C	2.464065	2.401505	−2.491778
C	1.993517	1.542475	−3.437708
C	−1.540659	−1.978635	3.435801
C	−2.399324	−2.451781	2.491118
H	3.128618	3.249072	2.621828
H	2.184406	1.545828	4.505746
H	−1.547184	−2.162828	−4.504863
H	−3.249087	−3.113174	−2.622088
H	3.128618	3.249072	−2.621828
H	−1.547184	−2.162828	4.504863
H	2.184406	1.545828	−4.505746
H	−3.249087	−3.113174	2.622088
C	3.065162	3.813586	0.000000
C	0.474516	−0.473018	4.909570
C	0.474516	−0.473018	−4.909570
C	−3.792088	−3.089294	0.000000
C	4.468181	3.804496	0.000000
C	2.394594	5.047195	0.000000
C	1.323313	−1.427760	−5.490101
C	−0.270369	0.371233	−5.748890
C	1.323313	−1.427760	5.490101
C	−0.270369	0.371233	5.748890
C	−3.750122	−4.492082	0.000000
C	−5.041209	−2.448446	0.000000
C	5.185351	5.004307	0.000000
C	3.111859	6.246204	0.000000
C	1.428442	−1.533071	−6.880290
C	−0.164349	0.266389	−7.137934
C	1.428442	−1.533071	6.880290
C	−0.164349	0.266389	7.137934

C	−4.932368	−5.237867	0.000000
C	−6.222718	−3.194809	0.000000
C	4.509283	6.227424	0.000000
C	0.685794	−0.685922	7.707364
C	0.685794	−0.685922	−7.707364
C	−6.171208	−4.591273	0.000000
H	4.996227	2.849687	0.000000
H	1.303688	5.060630	0.000000
H	1.904792	−2.088072	−4.844615
H	−0.935186	1.113991	−5.305193
H	1.904792	−2.088072	4.844615
H	−0.935186	1.113991	5.305193
H	−2.783425	−4.997895	0.000000
H	−5.081016	−1.358413	0.000000
H	6.276089	4.982248	0.000000
H	2.576189	7.196753	0.000000
H	2.094353	−2.278768	−7.317160
H	−0.748866	0.929990	−7.777346
H	2.094353	−2.278768	7.317160
H	−0.748866	0.929990	7.777346
H	−4.884095	−6.327823	0.000000
H	−7.185961	−2.682222	0.000000
H	5.069785	7.163368	0.000000
H	0.766436	−0.767849	8.792274
H	0.766436	−0.767849	−8.792274
H	−7.093512	−5.174059	0.000000
Cu	1.753919	−1.794877	0.000000
N	−1.246721	1.288298	0.000000
O	−2.413929	1.092305	0.000000

Table S22. Optimized coordinates (Å) of the LS Ag–CoTPP–NO (C_s) cluster at the SR-ZORA, BP86, TZP, spin-unrestricted level of theory.

Co	−0.002816	−0.003172	0.000000
N	1.086575	0.894853	1.397380
N	−0.888962	−1.077644	−1.393360
N	1.086575	0.894853	−1.397380
N	−0.888962	−1.077644	1.393360
C	1.900988	2.000504	1.224430
C	1.106098	0.633650	2.757881
C	−0.626664	−1.095209	−2.754559
C	−1.993720	−1.895586	−1.223232
C	1.900988	2.000504	−1.224430
C	1.106098	0.633650	−2.757881
C	−0.626664	−1.095209	2.754559
C	−1.993720	−1.895586	1.223232
C	2.245893	2.578007	0.000000
C	0.338271	−0.330551	−3.412939
C	0.338271	−0.330551	3.412939
C	−2.568802	−2.245534	0.000000
C	2.433266	2.434375	2.492320
C	−1.562445	−1.955699	−3.433130
C	1.966586	1.570253	3.435772
C	−2.425953	−2.426375	−2.491540
C	2.433266	2.434375	−2.492320
C	1.966586	1.570253	−3.435772
C	−1.562445	−1.955699	3.433130
C	−2.425953	−2.426375	2.491540
H	3.099929	3.280114	2.624033
H	2.163036	1.566392	4.502796
H	−1.558724	−2.148715	−4.500768
H	−3.270327	−3.094260	−2.624377
H	3.099929	3.280114	−2.624033
H	−1.558724	−2.148715	4.500768
H	2.163036	1.566392	−4.502796
H	−3.270327	−3.094260	2.624377
C	3.080909	3.822911	0.000000
C	0.500530	−0.499146	4.893698
C	0.500530	−0.499146	−4.893698
C	−3.807613	−3.089566	0.000000
C	4.483062	3.759579	0.000000
C	2.460337	5.082084	0.000000
C	1.412604	−1.436499	−5.402810
C	−0.252766	0.270983	−5.793552
C	1.412604	−1.436499	5.402810
C	−0.252766	0.270983	5.793552
C	−3.730903	−4.491112	0.000000
C	−5.072275	−2.480491	0.000000
C	5.247940	4.929570	0.000000
C	3.224695	6.252179	0.000000
C	1.570480	−1.598155	−6.782268
C	−0.095699	0.108427	−7.172675
C	1.570480	−1.598155	6.782268
C	−0.095699	0.108427	7.172675

C	−4.893771	−5.266664	0.000000
C	−6.234894	−3.256327	0.000000
C	4.620966	6.179600	0.000000
C	0.816944	−0.825799	7.670571
C	0.816944	−0.825799	−7.670571
C	−6.149191	−4.651036	0.000000
H	4.973649	2.784983	0.000000
H	1.370897	5.139102	0.000000
H	1.999804	−2.039825	−4.709118
H	−0.964448	1.001240	−5.405581
H	1.999804	−2.039825	4.709118
H	−0.964448	1.001240	5.405581
H	−2.751915	−4.972770	0.000000
H	−5.139338	−1.391828	0.000000
H	6.337008	4.862839	0.000000
H	2.726736	7.223000	0.000000
H	2.284104	−2.330383	−7.163076
H	−0.687430	0.715529	−7.859635
H	2.284104	−2.330383	7.163076
H	−0.687430	0.715529	7.859635
H	−4.817547	−6.355140	0.000000
H	−7.210537	−2.767918	0.000000
H	5.217680	7.092753	0.000000
H	0.940386	−0.951498	8.747376
H	0.940386	−0.951498	−8.747376
H	−7.056934	−5.255970	0.000000
Ag	1.899028	−1.934147	0.000000
N	−1.273973	1.332476	0.000000
O	−2.440123	1.130829	0.000000

Table S23. Optimized coordinates (Å) of the LS Au–CoTPP–NO (C_s) cluster at the SR-ZORA, BP86, TZP, spin-unrestricted level of theory.

Co	0.047938	−0.037912	0.000000
N	1.104568	0.884787	1.394744
N	−0.852622	−1.104130	−1.393685
N	1.104568	0.884787	−1.394744
N	−0.852622	−1.104130	1.393685
C	1.888493	2.010790	1.224072
C	1.150359	0.599494	2.749156
C	−0.567309	−1.145076	−2.747238
C	−1.972115	−1.897863	−1.223942
C	1.888493	2.010790	−1.224072
C	1.150359	0.599494	−2.749156
C	−0.567309	−1.145076	2.747238
C	−1.972115	−1.897863	1.223942
C	2.211134	2.602748	0.000000
C	0.407991	−0.387826	−3.400540
C	0.407991	−0.387826	3.400540
C	−2.562162	−2.224716	0.000000
C	2.424162	2.441261	2.491934
C	−1.507537	−2.000994	−3.426217
C	1.997615	1.547936	3.427521
C	−2.396585	−2.437751	−2.491349
C	2.424162	2.441261	−2.491934
C	1.997615	1.547936	−3.427521
C	−1.507537	−2.000994	3.426217
C	−2.396585	−2.437751	2.491349
H	3.069298	3.302914	2.627578
H	2.211762	1.531523	4.491145
H	−1.491684	−2.211089	−4.490579
H	−3.252293	−3.090334	−2.627856
H	3.069298	3.302914	−2.627578
H	−1.491684	−2.211089	4.490579
H	2.211762	1.531523	−4.491145
H	−3.252293	−3.090334	2.627856
C	3.006739	3.872770	0.000000
C	0.604261	−0.588749	4.872517
C	0.604261	−0.588749	−4.872517
C	−3.829817	−3.024558	0.000000
C	4.410231	3.853252	0.000000
C	2.346421	5.111536	0.000000
C	1.490319	−1.573672	−5.335346
C	−0.091267	0.192601	−5.808720
C	1.490319	−1.573672	5.335346
C	−0.091267	0.192601	5.808720
C	−3.807091	−4.428105	0.000000
C	−5.069908	−2.366638	0.000000
C	5.137315	5.047043	0.000000
C	3.073316	6.305197	0.000000
C	1.680135	−1.770613	−6.706018
C	0.098031	−0.005650	−7.179072
C	1.680135	−1.770613	6.706018
C	0.098031	−0.005650	7.179072

C	−4.999541	−5.157462	0.000000
C	−6.261900	−3.096330	0.000000
C	4.471123	6.276380	0.000000
C	0.984611	−0.987332	7.631080
C	0.984611	−0.987332	−7.631080
C	−6.230206	−4.493539	0.000000
H	4.931020	2.894577	0.000000
H	1.255724	5.134393	0.000000
H	2.032810	−2.184457	−4.612283
H	−0.785346	0.957226	−5.456526
H	2.032810	−2.184457	4.612283
H	−0.785346	0.957226	5.456526
H	−2.847495	−4.947172	0.000000
H	−5.094751	−1.276150	0.000000
H	6.228100	5.015275	0.000000
H	2.544895	7.259711	0.000000
H	2.373713	−2.539156	−7.050921
H	−0.448813	0.608337	−7.896513
H	2.373713	−2.539156	7.050921
H	−0.448813	0.608337	7.896513
H	−4.965799	−6.248138	0.000000
H	−7.217737	−2.570083	0.000000
H	5.039075	7.207929	0.000000
H	1.131940	−1.140296	8.701257
H	1.131940	−1.140296	−8.701257
H	−7.160715	−5.063160	0.000000
Au	1.870466	−1.911491	0.000000
N	−1.275848	1.244597	0.000000
O	−2.439789	1.049564	0.000000

Table S24. Optimized coordinates (Å) of the LS Cu–CoTPP–NO_2 (C_{2v}) cluster at the SR-ZORA, BP86, TZP, spin-unrestricted level of theory.

Co	0.000000	0.000000	−0.003873
N	1.388254	1.395112	0.137846
N	−1.388254	−1.395112	0.137846
N	1.388254	−1.395112	0.137846
N	−1.388254	1.395112	0.137846
C	2.751970	1.222626	−0.040539
C	1.221569	2.761233	0.325678
C	−1.221569	−2.761233	0.325678
C	−2.751970	−1.222626	−0.040539
C	2.751970	−1.222626	−0.040539
C	1.221569	−2.761233	0.325678
C	−1.221569	2.761233	0.325678
C	−2.751970	1.222626	−0.040539
C	3.405998	0.000000	−0.187079
C	0.000000	−3.420926	0.455979
C	0.000000	3.420926	0.455979
C	−3.405998	0.000000	−0.187079
C	3.437249	2.486902	0.037339
C	−2.493586	−3.435188	0.289340
C	2.493586	3.435188	0.289340
C	−3.437249	−2.486902	0.037339
C	3.437249	−2.486902	0.037339
C	2.493586	−3.435188	0.289340
C	−2.493586	3.435188	0.289340
C	−3.437249	2.486902	0.037339
H	4.509196	2.613481	−0.070240
H	2.628773	4.503133	0.424194
H	−2.628773	−4.503133	0.424194
H	−4.509196	−2.613481	−0.070240
H	4.509196	−2.613481	−0.070240
H	−2.628773	4.503133	0.424194
H	2.628773	−4.503133	0.424194
H	−4.509196	2.613481	−0.070240
C	4.879437	0.000000	−0.456593
C	0.000000	4.903337	0.675325
C	0.000000	−4.903337	0.675325
C	−4.879437	0.000000	−0.456593
C	5.810866	0.000000	0.592865
C	5.344761	0.000000	−1.780951
C	0.000000	−5.428008	1.976932
C	0.000000	−5.788737	−0.413520
C	0.000000	5.428008	1.976932
C	0.000000	5.788737	−0.413520
C	−5.810866	0.000000	0.592865
C	−5.344761	0.000000	−1.780951
C	7.182324	0.000000	0.323478
C	6.716714	0.000000	−2.048378
C	0.000000	−6.810052	2.185090
C	0.000000	−7.170444	−0.204408
C	0.000000	6.810052	2.185090
C	0.000000	7.170444	−0.204408

C	−7.182324	0.000000	0.323478
C	−6.716714	0.000000	−2.048378
C	7.638177	0.000000	−0.997644
C	0.000000	7.684365	1.095150
C	0.000000	−7.684365	1.095150
C	−7.638177	0.000000	−0.997644
H	5.455344	0.000000	1.624539
H	4.622915	0.000000	−2.598665
H	0.000000	−4.746574	2.829088
H	0.000000	−5.387192	−1.427942
H	0.000000	4.746574	2.829088
H	0.000000	5.387192	−1.427942
H	−5.455344	0.000000	1.624539
H	−4.622915	0.000000	−2.598665
H	7.895672	0.000000	1.149066
H	7.065171	0.000000	−3.082340
H	0.000000	−7.203493	3.202738
H	0.000000	−7.846799	−1.060472
H	0.000000	7.203493	3.202738
H	0.000000	7.846799	−1.060472
H	−7.895672	0.000000	1.149066
H	−7.065171	0.000000	−3.082340
H	8.708841	0.000000	−1.207320
H	0.000000	8.763160	1.257670
H	0.000000	−8.763160	1.257670
H	−8.708841	0.000000	−1.207320
Cu	0.000000	0.000000	2.351666
N	0.000000	0.000000	−1.975007
O	−1.094196	0.000000	−2.554599
O	1.094196	0.000000	−2.554599

Table S25. Optimized coordinates (Å) of the LS Ag–CoTPP–NO_2 (C_{2v}) cluster at the SR-ZORA, BP86, TZP, spin-unrestricted level of theory.

Co	0.000000	0.000000	0.039406
N	1.388130	1.393318	0.176099
N	−1.388130	−1.393318	0.176099
N	1.388130	−1.393318	0.176099
N	−1.388130	1.393318	0.176099
C	2.748248	1.222452	−0.028200
C	1.221523	2.758971	0.366231
C	−1.221523	−2.758971	0.366231
C	−2.748248	−1.222452	−0.028200
C	2.748248	−1.222452	−0.028200
C	1.221523	−2.758971	0.366231
C	−1.221523	2.758971	0.366231
C	−2.748248	1.222452	−0.028200
C	3.399466	0.000000	−0.193177
C	0.000000	−3.418430	0.499323
C	0.000000	3.418430	0.499323
C	−3.399466	0.000000	−0.193177
C	3.432284	2.487882	0.037030
C	−2.492355	−3.433927	0.310777
C	2.492355	3.433927	0.310777
C	−3.432284	−2.487882	0.037030
C	3.432284	−2.487882	0.037030
C	2.492355	−3.433927	0.310777
C	−2.492355	3.433927	0.310777
C	−3.432284	2.487882	0.037030
H	4.501374	2.616888	−0.093347
H	2.628669	4.501938	0.443610
H	−2.628669	−4.501938	0.443610
H	−4.501374	−2.616888	−0.093347
H	4.501374	−2.616888	−0.093347
H	−2.628669	4.501938	0.443610
H	2.628669	−4.501938	0.443610
H	−4.501374	2.616888	−0.093347
C	4.861567	0.000000	−0.518878
C	0.000000	4.904241	0.697362
C	0.000000	−4.904241	0.697362
C	−4.861567	0.000000	−0.518878
C	5.838908	0.000000	0.487683
C	5.267944	0.000000	−1.862732
C	0.000000	−5.452200	1.989133
C	0.000000	−5.770975	−0.406865
C	0.000000	5.452200	1.989133
C	0.000000	5.770975	−0.406865
C	−5.838908	0.000000	0.487683
C	−5.267944	0.000000	−1.862732
C	7.197121	0.000000	0.157314
C	6.626043	0.000000	−2.191573
C	0.000000	−6.838090	2.172880
C	0.000000	−7.156203	−0.223056
C	0.000000	6.838090	2.172880
C	0.000000	7.156203	−0.223056

C	−7.197121	0.000000	0.157314
C	−6.626043	0.000000	−2.191573
C	7.594102	0.000000	−1.183424
C	0.000000	7.693654	1.068008
C	0.000000	−7.693654	1.068008
C	−7.594102	0.000000	−1.183424
H	5.530265	0.000000	1.534269
H	4.509832	0.000000	−2.646875
H	0.000000	−4.785718	2.852843
H	0.000000	−5.351866	−1.414129
H	0.000000	4.785718	2.852843
H	0.000000	5.351866	−1.414129
H	−5.530265	0.000000	1.534269
H	−4.509832	0.000000	−2.646875
H	7.946462	0.000000	0.950451
H	6.927581	0.000000	−3.240146
H	0.000000	−7.249752	3.183356
H	0.000000	−7.816256	−1.091751
H	0.000000	7.249752	3.183356
H	0.000000	7.816256	−1.091751
H	−7.946462	0.000000	0.950451
H	−6.927581	0.000000	−3.240146
H	8.654109	0.000000	−1.441339
H	0.000000	8.774951	1.212215
H	0.000000	−8.774951	1.212215
H	−8.654109	0.000000	−1.441339
Ag	0.000000	0.000000	2.641294
N	0.000000	0.000000	−1.910308
O	−1.094088	0.000000	−2.488689
O	1.094088	0.000000	−2.488689

Table S26. Optimized coordinates (Å) of the LS Au–CoTPP–NO_2 (C_{2v}) cluster at the SR-ZORA, BP86, TZP, spin-unrestricted level of theory.

Co	0.000000	0.000000	0.084855
N	−1.392941	1.387005	0.195781
N	1.392941	−1.387005	0.195781
N	1.392941	1.387005	0.195781
N	−1.392941	−1.387005	0.195781
C	−1.223675	2.741653	−0.029630
C	−2.752510	1.222470	0.416037
C	2.752510	−1.222470	0.416037
C	1.223675	−2.741653	−0.029630
C	1.223675	2.741653	−0.029630
C	2.752510	1.222470	0.416037
C	−2.752510	−1.222470	0.416037
C	−1.223675	−2.741653	−0.029630
C	0.000000	3.387776	−0.212237
C	3.407408	0.000000	0.567925
C	−3.407408	0.000000	0.567925
C	0.000000	−3.387776	−0.212237
C	−2.487669	3.428215	0.048124
C	3.428164	−2.493049	0.357169
C	−3.428164	2.493049	0.357169
C	2.487669	−3.428215	0.048124
C	2.487669	3.428215	0.048124
C	3.428164	2.493049	0.357169
C	−3.428164	−2.493049	0.357169
C	−2.487669	−3.428215	0.048124
H	−2.618567	4.495445	−0.095197
H	−4.492893	2.631839	0.511348
H	4.492893	−2.631839	0.511348
H	2.618567	−4.495445	−0.095197
H	2.618567	4.495445	−0.095197
H	−4.492893	−2.631839	0.511348
H	4.492893	2.631839	0.511348
H	−2.618567	−4.495445	−0.095197
C	0.000000	4.844052	−0.560709
C	−4.882201	0.000000	0.830611
C	4.882201	0.000000	0.830611
C	0.000000	−4.844052	−0.560709
C	0.000000	5.836492	0.430959
C	0.000000	5.227654	−1.911242
C	5.362342	0.000000	2.149337
C	5.802807	0.000000	−0.228494
C	−5.362342	0.000000	2.149337
C	−5.802807	0.000000	−0.228494
C	0.000000	−5.836492	0.430959
C	0.000000	−5.227654	−1.911242
C	0.000000	7.189055	0.078260
C	0.000000	6.580221	−2.261829
C	6.736882	0.000000	2.402977
C	7.176749	0.000000	0.026259
C	−6.736882	0.000000	2.402977
C	−7.176749	0.000000	0.026259

C	0.000000	−7.189055	0.078260
C	0.000000	−6.580221	−2.261829
C	0.000000	7.564397	−1.268695
C	−7.647347	0.000000	1.342832
C	7.647347	0.000000	1.342832
C	0.000000	−7.564397	−1.268695
H	0.000000	5.544271	1.482112
H	0.000000	4.457113	−2.683163
H	4.652001	0.000000	2.977145
H	5.436057	0.000000	−1.255971
H	−4.652001	0.000000	2.977145
H	−5.436057	0.000000	−1.255971
H	0.000000	−5.544271	1.482112
H	0.000000	−4.457113	−2.683163
H	0.000000	7.951122	0.859191
H	0.000000	6.864610	−3.315123
H	7.096255	0.000000	3.433127
H	7.880686	0.000000	−0.807491
H	−7.096255	0.000000	3.433127
H	−7.880686	0.000000	−0.807491
H	0.000000	−7.951122	0.859191
H	0.000000	−6.864610	−3.315123
H	0.000000	8.620257	−1.543216
H	−8.719993	0.000000	1.541761
H	8.719993	0.000000	1.541761
H	0.000000	−8.620257	−1.543216
Au	0.000000	0.000000	2.642474
N	0.000000	0.000000	−1.866562
O	0.000000	−1.093480	−2.445608
O	0.000000	1.093480	−2.445608

Table S27. Optimized coordinates (Å) of the high spin (HS) Cu–CoTPP–O_2 (C_s) cluster at the SR-ZORA, BP86, TZP, spin-unrestricted level of theory.

Co	0.061713	−0.071390	0.000000
N	1.072846	0.881547	1.389910
N	−0.843638	−1.125220	−1.389732
N	1.072846	0.881547	−1.389910
N	−0.843638	−1.125220	1.389732
C	1.861696	2.009445	1.222374
C	1.106404	0.616759	2.752279
C	−0.578456	−1.153821	−2.750923
C	−1.961342	−1.925534	−1.222125
C	1.861696	2.009445	−1.222374
C	1.106404	0.616759	−2.752279
C	−0.578456	−1.153821	2.750923
C	−1.961342	−1.925534	1.222125
C	2.195173	2.594096	0.000000
C	0.372685	−0.372906	−3.408355
C	0.372685	−0.372906	3.408355
C	−2.549938	−2.255163	0.000000
C	2.381800	2.454873	2.489822
C	−1.521853	−2.005310	−3.429410
C	1.942143	1.574405	3.430519
C	−2.397363	−2.455722	−2.488879
C	2.381800	2.454873	−2.489822
C	1.942143	1.574405	−3.430519
C	−1.521853	−2.005310	3.429410
C	−2.397363	−2.455722	2.488879
H	3.022636	3.319813	2.623421
H	2.140461	1.573722	4.497157
H	−1.521839	−2.197161	−4.497236
H	−3.257798	−3.102660	−2.622462
H	3.022636	3.319813	−2.623421
H	−1.521839	−2.197161	4.497236
H	2.140461	1.573722	−4.497157
H	−3.257798	−3.102660	2.622462
C	3.008067	3.852258	0.000000
C	0.541586	−0.530448	4.889058
C	0.541586	−0.530448	−4.889058
C	−3.822660	−3.045435	0.000000
C	4.410634	3.807061	0.000000
C	2.369753	5.102303	0.000000
C	1.398239	−1.514611	−5.405377
C	−0.143347	0.308347	−5.782742
C	1.398239	−1.514611	5.405377
C	−0.143347	0.308347	5.782742
C	−3.813080	−4.448620	0.000000
C	−5.056223	−2.374803	0.000000
C	5.159292	4.987171	0.000000
C	3.119054	6.282065	0.000000
C	1.571212	−1.654353	−6.785582
C	0.028863	0.167827	−7.162136
C	1.571212	−1.654353	6.785582
C	0.028863	0.167827	7.162136

C	−5.012596	−5.166545	0.000000
C	−6.254498	−3.093635	0.000000
C	4.515241	6.227434	0.000000
C	0.887476	−0.812539	7.667177
C	0.887476	−0.812539	−7.667177
C	−6.235928	−4.490860	0.000000
H	4.914025	2.839075	0.000000
H	1.279664	5.144708	0.000000
H	1.933921	−2.170801	−4.717346
H	−0.812618	1.075005	−5.388964
H	1.933921	−2.170801	4.717346
H	−0.812618	1.075005	5.388964
H	−2.858715	−4.977414	0.000000
H	−5.068234	−1.284150	0.000000
H	6.249231	4.936102	0.000000
H	2.608515	7.246308	0.000000
H	2.243217	−2.422392	−7.171351
H	−0.510844	0.826339	−7.844763
H	2.243217	−2.422392	7.171351
H	−0.510844	0.826339	7.844763
H	−4.990168	−6.257413	0.000000
H	−7.205335	−2.558444	0.000000
H	5.100178	7.148299	0.000000
H	1.021544	−0.919870	8.744799
H	1.021544	−0.919870	−8.744799
H	−7.171706	−5.051824	0.000000
Cu	1.696250	−1.715367	0.000000
O	−1.373899	1.345554	0.000000
O	−2.618434	1.053211	0.000000

Table S28. Optimized coordinates (Å) of the HS Ag–CoTPP–O_2 (C_s) cluster at the SR-ZORA, BP86, TZP, spin-unrestricted level of theory.

Co	0.029751	−0.037609	0.000000
N	1.052582	0.908182	1.390836
N	−0.872009	−1.096478	−1.390113
N	1.052582	0.908182	−1.390836
N	−0.872009	−1.096478	1.390113
C	1.847054	2.031860	1.222930
C	1.083046	0.644282	2.753167
C	−0.607131	−1.122860	−2.751247
C	−1.984540	−1.904357	−1.222415
C	1.847054	2.031860	−1.222930
C	1.083046	0.644282	−2.753167
C	−0.607131	−1.122860	2.751247
C	−1.984540	−1.904357	1.222415
C	2.186060	2.612952	0.000000
C	0.345948	−0.343539	−3.408219
C	0.345948	−0.343539	3.408219
C	−2.569598	−2.239175	0.000000
C	2.365533	2.477548	2.491536
C	−1.545221	−1.980790	−3.429953
C	1.919686	1.600336	3.432500
C	−2.417787	−2.436703	−2.489473
C	2.365533	2.477548	−2.491536
C	1.919686	1.600336	−3.432500
C	−1.545221	−1.980790	3.429953
C	−2.417787	−2.436703	2.489473
H	3.007386	3.341888	2.626109
H	2.113261	1.600847	4.500028
H	−1.543250	−2.174093	−4.497497
H	−3.272789	−3.090685	−2.623451
H	3.007386	3.341888	−2.626109
H	−1.543250	−2.174093	4.497497
H	2.113261	1.600847	−4.500028
H	−3.272789	−3.090685	2.623451
C	3.010939	3.863781	0.000000
C	0.514503	−0.510797	4.887665
C	0.514503	−0.510797	−4.887665
C	−3.836828	−3.038168	0.000000
C	4.413138	3.805161	0.000000
C	2.385139	5.120210	0.000000
C	1.397661	−1.475177	−5.396882
C	−0.211105	0.285779	−5.787329
C	1.397661	−1.475177	5.396882
C	−0.211105	0.285779	5.787329
C	−3.819449	−4.441330	0.000000
C	−5.073952	−2.374380	0.000000
C	5.173774	4.977575	0.000000
C	3.145952	6.292686	0.000000
C	1.556808	−1.636404	−6.776179
C	−0.053826	0.123176	−7.166077
C	1.556808	−1.636404	6.776179
C	−0.053826	0.123176	7.166077

C	−5.014912	−5.165809	0.000000
C	−6.268377	−3.099557	0.000000
C	4.541636	6.224856	0.000000
C	0.831769	−0.836877	7.664151
C	0.831769	−0.836877	−7.664151
C	−6.242011	−4.496847	0.000000
H	4.907103	2.832317	0.000000
H	1.295546	5.173179	0.000000
H	1.963434	−2.099732	−4.703707
H	−0.903376	1.034265	−5.398780
H	1.963434	−2.099732	4.703707
H	−0.903376	1.034265	5.398780
H	−2.862176	−4.964977	0.000000
H	−5.091612	−1.283892	0.000000
H	6.263082	4.915407	0.000000
H	2.644991	7.261934	0.000000
H	2.248856	−2.388892	−7.157277
H	−0.626611	0.747898	−7.853336
H	2.248856	−2.388892	7.157277
H	−0.626611	0.747898	7.853336
H	−4.986402	−6.256570	0.000000
H	−7.222174	−2.569820	0.000000
H	5.134910	7.140247	0.000000
H	0.955122	−0.962060	8.740951
H	0.955122	−0.962060	−8.740951
H	−7.174545	−5.062858	0.000000
Ag	1.828158	−1.840807	0.000000
O	−1.364002	1.379624	0.000000
O	−2.611568	1.093454	0.000000

Table S29. Optimized coordinates (Å) of the HS Au–CoTPP–O_2 (C_s) cluster at the SR-ZORA, BP86, TZP, spin-unrestricted level of theory.

Co	0.028981	−0.044716	0.000000
N	1.036642	0.909186	1.388431
N	−0.909250	−1.063316	−1.390610
N	1.036642	0.909186	−1.388431
N	−0.909250	−1.063316	1.390610
C	1.805298	2.048624	1.223477
C	1.096663	0.618420	2.742954
C	−0.620283	−1.121635	−2.743610
C	−2.049073	−1.826732	−1.224111
C	1.805298	2.048624	−1.223477
C	1.096663	0.618420	−2.742954
C	−0.620283	−1.121635	2.743610
C	−2.049073	−1.826732	1.224111
C	2.120546	2.644808	0.000000
C	0.365804	−0.377337	−3.394018
C	0.365804	−0.377337	3.394018
C	−2.650344	−2.132817	0.000000
C	2.339153	2.481293	2.490754
C	−1.575640	−1.960530	−3.422686
C	1.931409	1.576133	3.423037
C	−2.481462	−2.365547	−2.489627
C	2.339153	2.481293	−2.490754
C	1.931409	1.576133	−3.423037
C	−1.575640	−1.960530	3.422686
C	−2.481462	−2.365547	2.489627
H	2.967852	3.354407	2.629193
H	2.146545	1.560719	4.486214
H	−1.560348	−2.176934	−4.485699
H	−3.356167	−2.992552	−2.626381
H	2.967852	3.354407	−2.629193
H	−1.560348	−2.176934	4.485699
H	2.146545	1.560719	−4.486214
H	−3.356167	−2.992552	2.626381
C	2.903761	3.921752	0.000000
C	0.585031	−0.594121	4.859919
C	0.585031	−0.594121	−4.859919
C	−3.934312	−2.904536	0.000000
C	4.307335	3.913938	0.000000
C	2.231470	5.153894	0.000000
C	1.549652	−1.516459	−5.293993
C	−0.160965	0.110075	−5.818000
C	1.549652	−1.516459	5.293993
C	−0.160965	0.110075	5.818000
C	−3.938158	−4.308054	0.000000
C	−5.160507	−2.221083	0.000000
C	5.023577	5.114053	0.000000
C	2.948324	6.353564	0.000000
C	1.762546	−1.732026	−6.658236
C	0.052081	−0.106374	−7.182231
C	1.762546	−1.732026	6.658236
C	0.052081	−0.106374	7.182231

C	−5.144422	−5.014069	0.000000
C	−6.366241	−2.927802	0.000000
C	4.345725	6.337150	0.000000
C	1.013994	−1.027912	7.605629
C	1.013994	−1.027912	−7.605629
C	−6.361885	−4.326092	0.000000
H	4.836734	2.959920	0.000000
H	1.140680	5.166617	0.000000
H	2.131751	−2.067088	−4.553944
H	−0.912148	0.828935	−5.487571
H	2.131751	−2.067088	4.553944
H	−0.912148	0.828935	5.487571
H	−2.989009	−4.845970	0.000000
H	−5.161989	−1.130414	0.000000
H	6.114556	5.092138	0.000000
H	2.412001	7.303677	0.000000
H	2.514265	−2.453900	−6.980874
H	−0.537734	0.445434	−7.915918
H	2.514265	−2.453900	6.980874
H	−0.537734	0.445434	7.915918
H	−5.132123	−6.105199	0.000000
H	−7.311480	−2.382871	0.000000
H	4.904716	7.273895	0.000000
H	1.179282	−1.197377	8.670708
H	1.179282	−1.197377	−8.670708
H	−7.303142	−4.877569	0.000000
Au	1.806568	−1.823624	0.000000
O	−1.351107	1.371639	0.000000
O	−2.601106	1.101476	0.000000

Table S30. Co Hirshfeld's charge (Q_{Co}, *e*); CoTPP, single atoms and small molecules *BE*s and ΔE_{sp}^{MS} (MS = Cu, Ag, Au); all the energy terms are in eV.

	CoTPP	Cu	Ag	Au	CO	NH_3	NO	NO_2	O_2
Q_{Co}	0.21								
ΔE_{sp}^{MS}		0.26	0.22	0.20					
BE	528.85				14.69	19.27	12.08	18.04	9.67

Table S31. Q_{Co} (*e*) and Q_{MS} (*e*) of the MS–CoTPP clusters; MS–Co bond lengths (BL, Å); MS–CoTPP *BE*s, CoTPP preparation energies (ΔE_{prep}^{CoTPP}), ΔE_{sp}^{MS} (see Table S30) and *BEs* of the MS–Co bond; all the energy terms are in eV.

	Cu–CoTPP	Ag–CoTPP	Au–CoTPP
Q_{Co}	0.04	0.04	0.08
Q_{MS}	0.19	0.24	0.05
$BL_{MS–Co}$	2.27	2.47	2.46
ΔE_{prep}^{CoTPP}	0.02	0.03	0.05
ΔE_{sp}^{MS}	0.26	0.22	0.20
BE	530.30	530.01	530.20
$BE_{MS–Co}$	1.17	0.91	1.11

Table S32. Q_{Co} (*e*) and $Q_{^0L}$ (*e*) of the LS CoTPP–^{0}L adducts; Nalewajski-Mrozek ($^{NM}I_{Co–D}$)[a] indexes; Co–D BL (Å);[a] ΔE_{prep}^{CoTPP}, $\Delta E_{prep}^{^0L}$ and *BE*s of the CoTPP–^{0}L adducts and of the Co–D bond;[a] all the energy terms are in eV.

	**CoTPP–CO**	**CoTPP–NH_3**
Q_{Co}	0.11	0.13
$Q_{^0L}$	0.01	0.21
$^{NM}I_{Co–D}$	0.94	0.41
$BL_{Co–D}$	1.859	2.194
ΔE_{prep}^{CoTPP}	0.28	0.10
$\Delta E_{prep}^{^0L}$	0.02	0.01
BE	544.17	548.49
$BE_{Co–D}$[b]	0.33	0.28

[a]D stands for the ^{0}L donor atom; [b]Co–^{0}L *BE*s ($BE_{Co–D}$) include both ΔE_{prep}^{CoTPP} and $\Delta E_{prep}^{^0L}$.

Table S33. Q_{Co} (*e*) and $Q_{^1L}$ (*e*) of the LS CoTPP–^{1}L adducts; $^{NM}I_{Co–D}$ indexes; Co–D BL (Å); ΔE_{prep}^{CoTPP}, $\Delta E_{prep}^{^1L}$ and *BE*s of the CoTPP–^{1}L adducts and of the Co–D bond; all the energy terms are in eV.

	**CoTPP–NO**[b]	**CoTPP–NO_2**[c]
Q_{Co}	0.13	0.14
$Q_{^1L}$	−0.02	−0.16
$^{NM}I_{Co–D}$	1.21	0.69
$BL_{Co–D}$	1.805	1.878
ΔE_{prep}^{CoTPP}	0.21	0.21
$\Delta E_{prep}^{^1L}$	0.02	0.11
BE	542.48	548.22
$BE_{Co–D}$[d]	1.32	1.03

[a]D stands for the ^{1}L donor atom; [b]The $Co-\widehat{N}-O$ bond angle (BA) is 122.5°; [c]The $O-\widehat{N}-O$ BA is 125.3°; [c]Co–^{1}L *BE*s ($BE_{Co–N}$) include both ΔE_{prep}^{CoTPP} and $\Delta E_{prep}^{^1L}$

Table S34. Q_{Co} (*e*) and $Q_{^2L}$ (*e*) of the LS CoTPP–^{2}L adducts; $^{NM}I_{Co–O}$ indexes; Co–O BL (Å); ΔE_{prep}^{CoTPP}, $\Delta E_{prep}^{^2L}$, *BE*s of the CoTPP–^{2}L adducts and of the Co–^{2}L bond; all the energy terms are in eV.

	**CoTPP–O_2**[a]
Q_{Co}	0.16
$Q_{^2L}$	−0.11
$^{NM}I_{Co–O}$	0.85
$BL_{Co–O}$	1.858
ΔE_{prep}^{CoTPP}	0.21
$\Delta E_{prep}^{^2L}$	0.08
BE	539.07
$BE_{Co–O}$[b]	0.26

[a]The $Co-\widehat{O}-O$ BA is 119.7°; [b]Co–^{2}L *BE* ($BE_{Co–O}$) includes both ΔE_{prep}^{CoTPP} and $\Delta E_{prep}^{^2L}$

Table S35. Co, MS and L formal oxidation states in the free CoTPP complex and in the MS–CoTPP, CoTPP–L, and MS–CoTPP–L clusters. .

	**-**	**CO**	**NH_3**	**NO**	**NO_2**	**O_2**
CoTPP	Co^{II}	CO+Co^{II}	NH_3+Co^{II}	$NO^{(+)}$+Co^{I}	$NO_2^{(-)}$+Co^{III}	$O_2^{(-)}$+Co^{III}
Cu–CoTPP	Co^{I}+Cu^{I}	CO+Co^{II}+Cu^0	NH_3+Co^{II}+Cu^0	$NO^{(+)}$+Co^{I}+Cu^0	$NO_2^{(-)}$+Co^{II}+Cu^0	$O_2^{(-)}$+Co^{III}+Cu^0
Ag–CoTPP	Co^{I}+Ag^{I}	CO+Co^{II}+Ag^0	NH_3+Co^{II}+Ag^0	$NO^{(+)}$+Co^{I}+Ag^0	$NO_2^{(-)}$+Co^{II}+Cu^0	$O_2^{(-)}$+Co^{III}+Ag^0
Au–CoTPP	Co^{II}+Au^0	CO+Co^{II}+Au^0	NH_3+Co^{II}+Au^0	$NO^{(+)}$+Co^{I}+Au^0	$NO_2^{(-)}$+Co^{II}+Au^0	$O_2^{(-)}$+Co^{III}+Au^0

Table S36. Q_{Co} (*e*), Q_{Cu} (*e*) and Q_L (*e*) of the Cu–CoTPP–L clusters; $^{NM}I_{Cu\text{–}Co}$ and $^{NM}I_{Co\text{–}D}$ indexes;[a] Co–D BLs (Å);[a] $\Delta E_{prep}^{CoTPP\text{–}L}$, $\Delta E_{prep}^{Cu\text{–}CoTPP}$, ΔE_{prep}^{L}, *BE*s of the Cu–CoTPP–L clusters and of the Co–L and Cu–Co bonds; all the energy terms are in eV.

	Cu–CoTPP–CO	Cu–CoTPP–NH$_3$	Cu–CoTPP–NO	Cu–CoTPP–NO$_2$	Cu–CoTPP–O$_2$
Q_{Co}	0.02	0.03	0.04	0.03	0.03
Q_{Cu}	0.09	0.09	0.07	0.17	0.18
Q_L	0.04	0.21	−0.004	−0.21	−0.10
$^{NM}I_{Cu\text{–}Co}$	0.84	1.00	0.28	0.35	0.55
$^{NM}I_{Co\text{–}D}$	0.20	0.24	0.97	0.50	0.48
$BL_{Cu\text{–}Co}$	2.343	2.305	2.442	2.356	2.318
$BL_{Co\text{–}D}$	1.883	2.198	1.860	1.971	2.017
$\Delta E_{prep}^{CoTPP\text{–}L}$	0.062	0.038	0.060	0.031	0.079
ΔE_{prep}^{L}	0.011	0.004	0.015	0.145	0.058
$\Delta E_{prep}^{Cu\text{–}CoTPP}$	0.240	0.060	0.293	0.218	0.109
$BE_{Cu\text{–}Co}$	0.79	1.03	0.41	0.68	0.77
$BE_{Co\text{–}D}$	0.04	0.20	0.52	0.50	0.05
BE	545.28	549.81	543.21	549.20	540.19

[a]D stands for the L donor atom.

Table S37. Q_{Co} (*e*), Q_{Ag} (*e*) and Q_L (*e*) of Ag–CoTPP-L clusters; $^{NM}I_{Ag\text{–}Co}$ and $^{NM}I_{Co\text{–}D}$ indexes; Co–D BLs (Å); $\Delta E_{prep}^{CoTPP\text{–}L}$, $\Delta E_{prep}^{Ag\text{–}CoTPP}$, ΔE_{prep}^{L}, *BE*s of the Ag–CoTPP–L clusters and of the Co–L and Ag–Co bonds; all the energy terms are in eV.

	Ag–CoTPP–CO	Ag–CoTPP–NH$_3$	Ag–CoTPP–NO	Ag–CoTPP–NO$_2$	Ag–CoTPP–O$_2$
Q_{Co}	0.03	0.03	0.05	0.04	0.04
Q_{Ag}	0.11	0.12	0.08	0.18	0.20
Q_L	0.04	0.21	0.004	−0.20	−0.11
$^{NM}I_{Ag\text{–}Co}$	0.81	0.97	0.20	0.29	0.47
$^{NM}I_{Co\text{–}D}$	0.71	0.24	1.04	0.54	0.54
$BL_{Ag\text{–}Co}$	2.569	2.518	2.710	2.602	2.547
$BL_{Co\text{–}D}$	1.877	2.208	1.844	1.950	1.988
$\Delta E_{prep}^{CoTPP\text{–}L}$	0.034	0.012	0.026	0.038	0.059
ΔE_{prep}^{L}	0.011	0.005	0.014	0.141	0.062
$\Delta E_{prep}^{Ag\text{–}CoTPP}$	0.255	0.071	0.345	0.273	0.132
$BE_{Ag\text{–}Co}$	0.55	0.74	0.26	0.40	0.54
$BE_{Co\text{–}D}$	0.01	0.10	0.54	0.43	0.02
BE	544.96	549.46	542.99	548.88	539.89

Table S38. Q_{Co} (*e*), Q_{Au} (*e*) and Q_L (*e*) of Au–CoTPP–L clusters; $^{NM}I_{Au\text{–}Co}$ and $^{NM}I_{Ag\text{–}D}$ indexes; Co–D BLs (Å); $\Delta E_{prep}^{CoTPP\text{–}L}$, $\Delta E_{prep}^{Au\text{–}CoTPP}$, ΔE_{prep}^{L}, *BE*s of the Au–CoTPP–L clusters and of the Co–L and Au–Co bonds; all the energy terms are in eV.

	Au–CoTPP–CO	Au–CoTPP–NH$_3$	Au–CoTPP–NO	Au–CoTPP–NO$_2$	Au–CoTPP–O$_2$
Q_{Co}	0.06	0.06	0.06	0.05	0.06
Q_{Au}	−0.07	−0.07	−0.02	0.03	0.04
Q_L	0.07	0.25	0.02	−0.19	−0.09
$^{NM}I_{Au\text{–}Co}$	0.85	1.06	0.39	0.37	0.60
$^{NM}I_{Co\text{–}D}$	0.78	0.34	0.97	0.55	0.53
$BL_{Au\text{–}Co}$	2.522	2.498	2.614	2.558	2.515
$BL_{Co\text{–}D}$	1.822	2.084	1.843	1.951	1.978
$\Delta E_{prep}^{CoTPP\text{–}L}$	0.140	0.092	0.058	0.038	0.089
ΔE_{prep}^{L}	0.009	0.001	0.010	0.143	0.059
$\Delta E_{prep}^{CoTPP\text{–}Au}$	0.267	0.074	0.346	0.301	0.162
$BE_{Au\text{–}Co}$	0.92	1.14	0.49	0.62	0.72
$BE_{Co\text{–}D}$	0.25	0.38	0.59	0.40	−0.01
BE	545.42	549.92	543.23	549.08	540.08

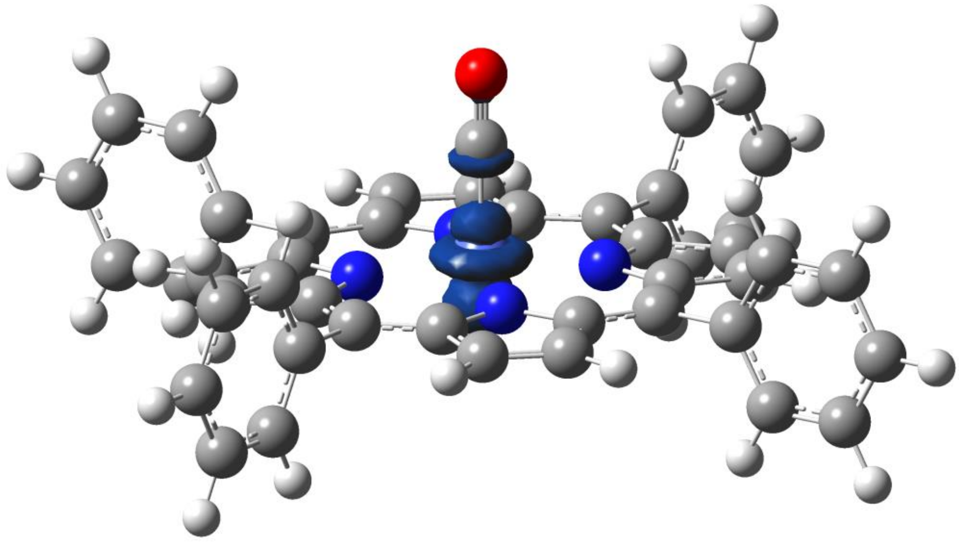

Figure S1. 3D plot of the CoTPP–CO spin density. The displayed isosurface corresponds to +0.005 $e \times Å^{-3}$ value.

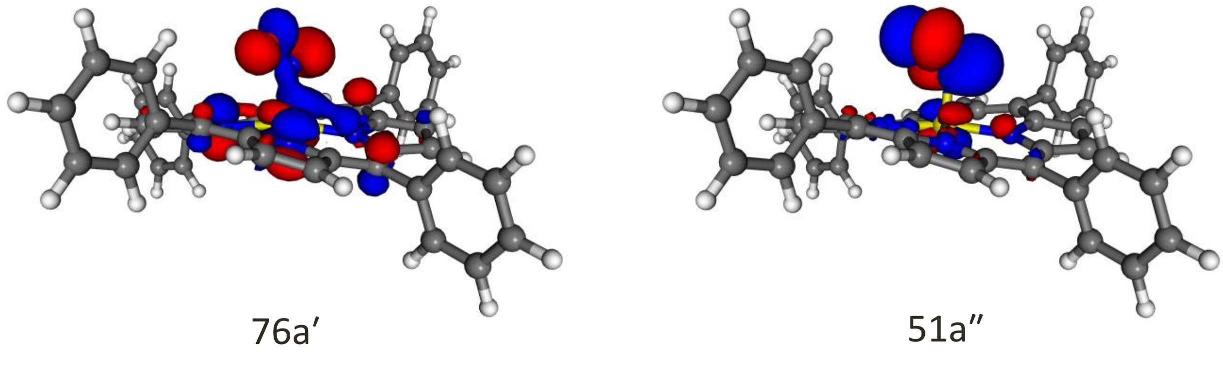

Figure S2. 3D plot of the CoTPP–NO 76a′ HOMO (left panel) and 51a″ LUMO (right panel). The displayed isosurfaces correspond to ±0.03 $e^{1/2} \times Å^{-3/2}$ values.

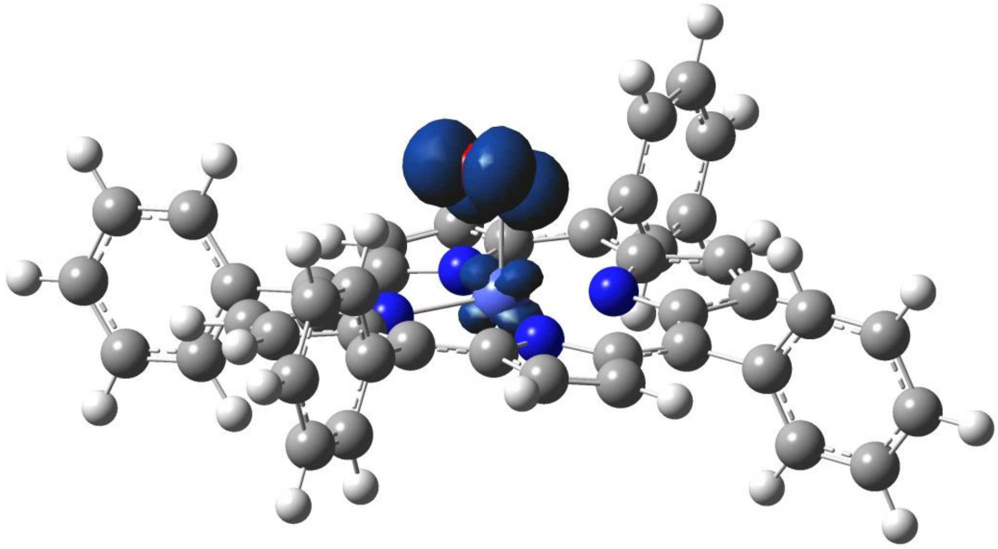

Figure S3. 3D plot of the CoTPP–(η^1-O_2) spin density. The displayed isosurface corresponds to +0.005 $e \times Å^{-3}$ value.
